# Serum metabolic and microbial profiling yields insights into promoting effect of tryptophan‐related metabolites for health longevity in centenarians

**DOI:** 10.1002/imt2.70025

**Published:** 2025-04-20

**Authors:** Xiaorou Qiu, Chao Mu, Jie Hu, Jiaxin Yu, Wenbo Tang, Yueli Liu, Yongmei Huang, Yixian Lu, Peihua Tang, Jingzhen Wu, Zixuan Huang, Xianlin Mei, Huaguo Xiang, Hao Lin, Yi Qi, Hui Luo, Xuemeng Li

**Affiliations:** ^1^ Zhanjiang Key Laboratory of Human Microecology and Clinical Translation Research, the Marine Biomedical Research Institute of Guangdong Zhanjiang Guangdong Medical University Zhanjiang China; ^2^ Dongguan Key Laboratory of Stem Cell and Regenerative Tissue Engineering, the First Dongguan Affiliated Hospital, School of Basic Medical Sciences Guangdong Medical University Dongguan China; ^3^ The Medical Laboratory Fuyong People's Hospital of Baoan District Shenzhen China; ^4^ Institute of Cancer Prevention and Treatment Harbin Medical University Harbin China; ^5^ Southern Marine Science and Engineering Guangdong Laboratory (Zhanjiang) Zhanjiang China; ^6^ Suixi County People's Hospital the Affiliated Hospital of Guangdong Medical University Zhanjiang China

**Keywords:** centenarians, multi‐omics analysis, serum metabolome, gut microbiomes, oral microbiomes, tryptophan metabolism, 5‐methoxyindoleacetic acid

## Abstract

A better understanding of the characteristic serum metabolites and microbiota from the gut and oral cavity in centenarians could contribute to elucidating the mutual connections among them and would help provide information to achieve healthy longevity. Here, we have recruited a total of 425 volunteers, including 145 centenarians in Suixi county — the first certified “International Longevity and Health Care Base” in China. An integrative analysis for the serum metabolites, gut, and oral microbiota of centenarians (aged 100–120) was compared with those of centenarians' lineal relatives (aged 24–86), the elderly (aged 65–88) and young (aged 23–54). Strikingly distinct metabolomic and microbiological profiles were observed within the centenarian signature, longevity family signature, and aging signature, underscoring the metabolic and microbiological diversity among centenarians and their lineal relatives. Within the centenarian between healthy and frail individuals, significant differences in metabolite profiles and microbiota compositions are observed, suggesting that healthy longevity is associated with unique metabolic and microbiota patterns. Through an integrative analysis, the tryptophan pathway has been revealed to be an important potential mechanism for individuals to achieve healthy longevity. Specifically, a key tryptophan metabolite, 5‐methoxyindoleacetic acid (5‐MIAA), was revealed to be associated with the genus *Christensenellaceae* R‐7 group, and it exhibited effects of delaying cell senescence, promoting lifespan, and alleviating inflammation. Our characterization of the extensive metabolomic and microbiota remodeling in centenarians may offer new scientific insights for achieving healthy longevity.

## Highlights

- Distinct metabolomic and microbiological profiles are observed in centenarian and longevity family. Healthy centenarians exhibit unique metabolic and microbiota patterns compared to frail counterparts.
- Tryptophan metabolism is enriched in both serum metabolites and gut microbiota of centenarians, potentially contributing to healthy longevity.
- A key tryptophan metabolite 5‐methoxyindoleacetic acid (5‐MIAA) is linked to the *Christensenellaceae* R‐7 group, and exhibits effects of delaying cell senescence, promoting lifespan and alleviating inflammation.

## Graphical abstract

Schematic representation of the design. Tryptophan metabolism is enriched in serum metabolites and gut microbiota of centenarians, potentially contributing to healthy longevity. A key tryptophan metabolite 5‐methoxyindoleacetic acid (5‐MIAA) is linked to the *Christensenellaceae* R‐7 group and exhibits effects of delaying cell senescence, promoting lifespan, and alleviating inflammation.

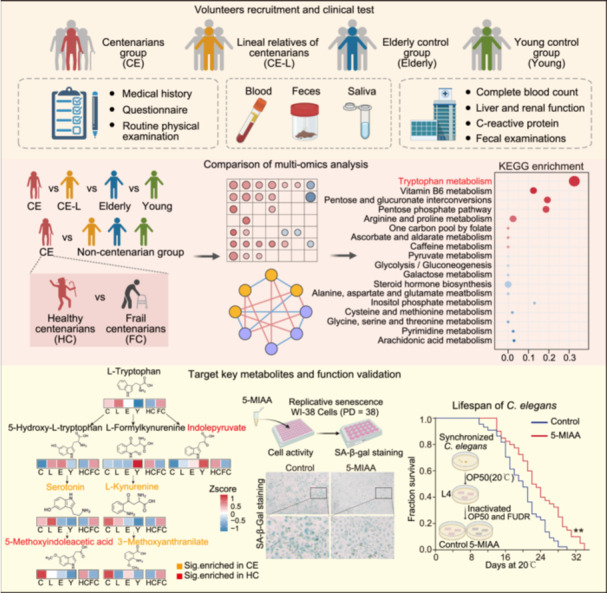

## INTRODUCTION

With the intensification of global aging, achieving healthy aging and extending lifespan have become significant focuses of biomedical research worldwide [1]. Centenarians, who exhibit extraordinary longevity and relatively good health status, are ideal subjects for unraveling the secrets of longevity [2], as they have reached the limits of human lifespan while largely escaping, delaying, or avoiding age‐related diseases [3]. Multi‐omics studies of centenarians, such as the integration of metabolomics and microbiomics, can elucidate key characteristics during the aging process and assist in identifying biomarkers of “healthy aging”. These biomarkers not only enhance our understanding of the aging process but also hold the potential for developing novel antiaging therapies [4].

In the study of centenarians, significant progress has been made in serum metabolomics. International research has shown that the levels of various metabolites in the serum of centenarians, such as amino acids and lipids, differ significantly from those in the general elderly population, and these metabolites may be associated with antioxidative and immune regulatory functions [5, 6]. In China, similar studies have identified distinctive metabolic characteristics in centenarians, potentially providing a molecular basis for their mental health and lower incidence of chronic diseases [7, 8]. Concurrently, studies on fecal microbiome have revealed that a highly diverse and stable gut microbiota in centenarians, which may promote longevity by maintaining immune function and resisting pathogenic [9]. In terms of oral microbiomics, a unique microbial composition exists in centenarians and may promote health by influencing both oral and systemic immune states [10, 11]. Recently, an increasing number of studies on multi‐omics and integrative analyses for centenarians have gradually begun to reveal the complex interactions between related factors in promoting healthy longevity [12, 13, 14]. Additionally, it has also been found that centenarians and their descendants share similarities in metabolic and microbiome profiles, while also exhibiting differences due to lifestyle and environmental factors [13, 14, 15, 16].

This study focuses on centenarians, their lineal relatives, elderly and young individuals from Suixi county, China. Utilizing comprehensive metabolomic and microbiomic approaches, including serum untarget metabolomics and 16S ribosomal RNA (16S rRNA) sequencing, we aim to uncover the biological mechanisms behind longevity. Through multi‐omics integrative analyses, we revealed that the tryptophan pathway may act as an important mechanism for individuals to achieve healthy longevity and identified a key tryptophan metabolite 5‐methoxyindoleacetic acid (5‐MIAA), which exhibited effects of delaying cell senescence, promoting lifespan, and alleviating inflammation.

## RESULTS

### Schematic diagram of study including centenarians and lineal relatives

**Figure 1:**
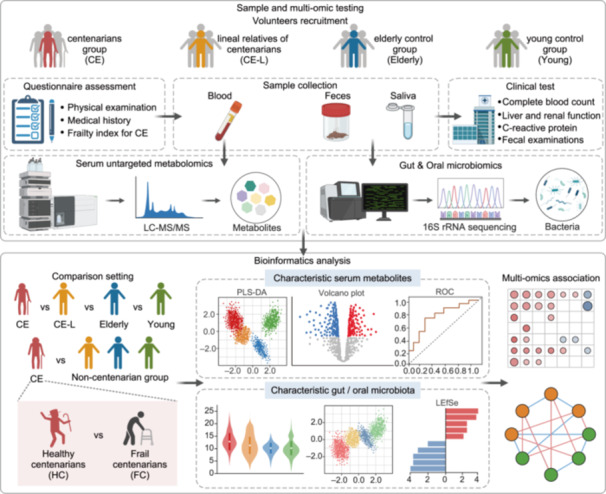
Schematic representation of the design to identify the characteristic biomarkers of centenarians through multi‐omics analysis for targeting the serum metabolome, the gut microbiome, and oral microbiome.

A total of 425 volunteers were recruited from Suixi county, Guangdong province, China, including 145 centenarians. These participants were divided into four groups: centenarians group (≥100 years old, CE), lineal relatives of the centenarians (24–86 years old, CE‐L), elderly control group (65–88 years old, Elderly), and young control group (23–54 years old, Young), as scheme shown in Figure 1. The medical history, questionnaire, and routine physical examination were conducted for all participants. Based on the previous results, the frailty index (FI) was calculated for the centenarian group, and then used to further distinguish between healthy centenarians (HC) and frail centenarians (FC). Clinical samples, containing blood, saliva, and feces were collected according to participants' preferences. Clinical tests were conducted on these samples, including complete blood count, liver function, renal function, C‐reactive protein, and routine fecal examinations. Then, laboratory examinations were performed for further study, including untargeted metabolomic techniques for serum metabolites, 16S rRNA sequencing for the gut microbiota in feces and the oral microbiota in saliva. The serum metabolites, gut and oral microbiota among various groups were analyzed to identify differential metabolites and bacterial genera. Finally, multi‐omics analyses were integrated to discover the serum metabolic characteristics of centenarians and their relationship with gut or oral microbiota.

### Characterization of the serum metabolomic signatures in centenarians

**Figure 2:**
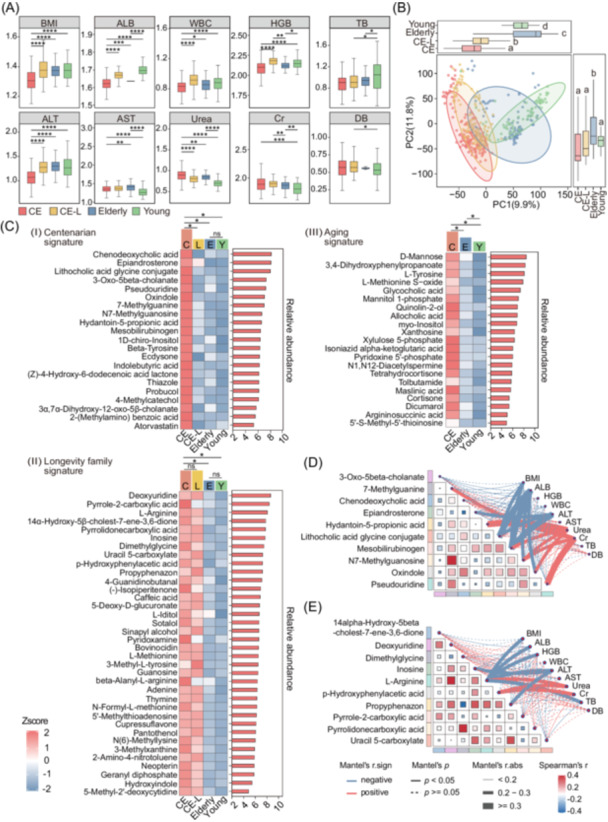
Untargeted metabolome analysis of serum metabolites for centenarians (CE), lineal relatives of the centenarians (CE‐L), and control group of Elderly and Young. (A) Box plots showed the difference across groups in clinical indicators, including body mass index (BMI), albumin (ALB), white blood cell count (WBC), hemoglobin (HGB), total bilirubin (TB), direct bilirubin (DB), alanine aminotransferase (ALT), aspartate aminotransferase (AST), Urea, and creatinine (Cr). (B) Partial least squares discriminant analysis (PLS‐DA) plot of serum metabolites for positive ion mode in CE, CE‐L, Elderly, and Young group. The overall distribution of principal component 1 (PC1) and principal component 2 (PC2) scores within each group were showed in the marginal box plots. No common letters within boxes indicated significant differences among distinct groups according to one‐way analysis of variance (ANOVA) with two‐sided Tukey's post hoc test (*p* < 0.05). (C) Changes in the relative abundance of serum metabolites among CE, CE‐L, Elderly and Young group were identified based on the following signatures: (Ⅰ) metabolites of the centenarian signature, with 21 metabolites significantly elevated in centenarians; (Ⅱ) longevity family characteristic metabolites, with 36 metabolites significantly elevated in long‐lived families compared to control groups of Elderly and Young; (Ⅲ) Age‐related metabolites, with 21 metabolites increased with age. The first feature (“centenarian signature”) included centenarian‐specific metabolites whose abundance was significantly different in centenarians compared to their lineal relatives and the control group of elderly and young with false discovery rate (FDR) < 0.05, while no statistical difference between the two control groups was observed. The second feature (“longevity family signature”) included metabolites whose abundance was similar in centenarians and their lineal relatives, but distinct from the elderly and young group. The third feature (“aging signature”) included metabolites whose abundance was increased or decreased with age. (D, E) Spearman's rank correlation analysis between clinical indicators mentioned in (A) and the top 10 metabolites with the centenarian signature (D), and the top 10 metabolites with longevity family signature (E). *p < 0.05; **p < 0.01; ***p < 0.001; ****p < 0.0001.

Blood samples were collected from all 425 participants for clinical indicator assessment and untargeted metabolomic analysis. The data and clinical indicators for this study cohort are detailed in Table S1. We observed that most health indices of centenarians were significantly reduced compared to those of the other three groups, such as body mass index (BMI), albumin (ALB), white blood cell count (WBC), hemoglobin (HGB), and alanine aminotransferase (ALT). The levels of aspartate aminotransferase (AST) in centenarians were higher than those in the elderly and young group. Besides, the levels of urea were observed to be higher in centenarians compared to the other groups, while the levels of creatinine (Cr) in centenarians were slightly higher than those in the young group. However, there were no significant differences observed in total bilirubin (TB) and direct bilirubin (DB) between the centenarians and the other groups (Figure 2A). These indicators have changed and deviated in centenarians, but they remain within a healthy reference range in the clinic. Therefore, the degree of change in these clinical indicators may be considered for establishing the reference ranges of clinical indicators for the unique population of long‐lived individuals, especially centenarians.

Subsequently, serum metabolites were analyzed using ultrahigh‐performance liquid chromatography‐tandem mass spectrometry (UHPLC‐MS), identifying 12,809 and 12,137 metabolites in positive and negative ion modes, respectively. 808 metabolites at metabolomics standards initiative level 2 confidence were annotated and confirmed through databases, including human metabolome database (HMDB), MassBank, LipidMaps, and mzCloud (Table S2). Partial least squares discriminant analysis (PLS‐DA) was utilized to elucidate the serum metabolic characteristics of the study cohort, with results for the positive ion mode (Figure 2B) and for the negative ion mode (Figure S1A). The PLS‐DA plot revealed a trend in the distribution of metabolites that shifts progressively from the young to centenarians with advancing age. At the principal component 1 (PC1) level, centenarians exhibited significant differences compared to the other three groups, suggesting the presence of distinct metabolites that characterize their unique metabolic profile. At the principal component 2 (PC2) level, no statistical differences were observed between centenarians and both their lineal relatives and young groups, indicating similar characteristics within longevity families (i.e., centenarians and their lineal relatives) that may also resemble those of young individuals.

To identify differential metabolites for centenarians and lineal relatives of the centenarians, metabolites were characterized by three distinct signatures [9]: (Ⅰ) the centenarian signature, (Ⅱ) the longevity family signature, and (Ⅲ) the aging signature. The centenarian signature encompasses metabolites unique to centenarians, which show significant abundance differences for centenarians in comparison with lineal relatives, the elderly and young control groups, but not between the two control groups of the elderly and young group. The longevity family signature includes similar abundance of metabolites between centenarians and their lineal relatives, but with significant abundance differences between centenarians and the two control groups. The aging signature comprises metabolites that vary with age, exhibiting significant differences in abundance among centenarians and the two control groups, with a trend of gradual increase or decrease. Following the above concepts, three distinct categories of metabolites were identified with significantly elevated abundance in the centenarian cohort: (Ⅰ) 21 centenarian‐signature metabolites, (Ⅱ) 36 metabolites with the longevity family signature, and (Ⅲ) 21 aging‐signature metabolites, as illustrated in Figure 2C. Conversely, those metabolites displaying a marked decreasing trend within the centenarian group are delineated in Figure S1B–D; and the enrichment of KEGG pathways was further analyzed between CE and the other three groups, respectively (Figure S2). Specifically, among the top 10 elevated centenarian‐signature metabolites, chenodeoxycholic acid, lithocholic acid glycine conjugate, and 3‐oxo‐5beta‐cholanate belong to the bile acid family, typically secondary bile acids produced by gut microbiota metabolism. Among the top 10 elevated metabolites with the longevity family signature, l‐arginine and dimethylglycine are known metabolites associated with the gut microbiota.

Subsequently, we conducted a Spearman's rank correlation analysis between the aforementioned top 10 metabolites significantly elevated in centenarians and the above‐mentioned clinical indicators shown in Figure 2A (Figure 2D). The analysis revealed that a significant number of metabolites increased in centenarians, such as 7‐methylguanine, hydantoin‐5‐propionic acid, mesobilirubinogen, N7‐methylguanosine, oxindole, and pseudouridine, exhibit positive correlations with urea and Cr levels. This finding implies a likely link between these centenarian‐specific metabolites and renal metabolic capacity, potentially underpinning the sustained renal function observed in this demographic. Similarly, Figure 2E illustrated that metabolites elevated in longevity families, notably l‐arginine and p‐hydroxyphenylacetic acid, also show significant positive correlations with urea and Cr levels, suggesting their potential involvement in renal metabolic processes. Although the exact mechanisms connecting these metabolites to renal metabolism in centenarians and longevity families remain to be fully elucidated, the observed associations highlighted the possible influence of these metabolites on overall health and longevity. This underscores the importance of further exploration into how these metabolic signatures might contribute to physiological resilience and sustained health in these unique populations.

### Overview of the microbiota signatures in gut and oral of centenarians

**Figure 3:**
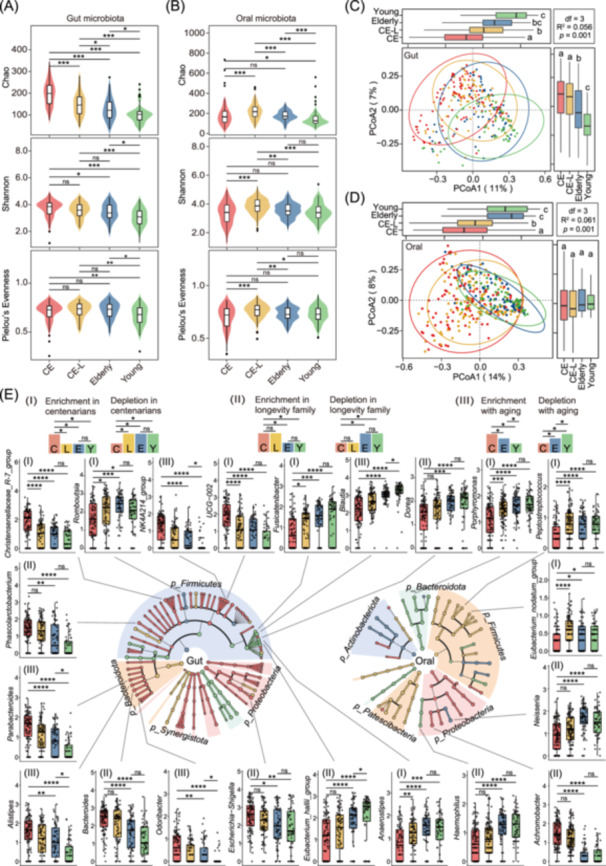
Analysis of gut and oral microbiota for diversity and characteristic genera for CE, CE‐L, and control group of Elderly and Young. (A–B) Alpha diversity of (A) gut microbiota and (B) oral microbiota were assessed using the Chao richness index, Shannon diversity index, and Pielou's evenness, respectively. (C–D) Principal Coordinate Analysis (PCoA) based on Bray–Curtis distances provide beta diversity analysis of (C) gut microbiota and (D) oral microbiota. The side box plots depict the overall distribution of PCoA1 and PCoA2 scores across different groups. Significant differences among groups, as determined by one‐way analysis of variance (ANOVA) followed by the two‐sided Tukey's post hoc test, are indicated by different letters (*p* < 0.05). (E) Significantly different characteristic genera in the gut and oral microbiota, including (Ⅰ) enrichment or depletion in centenarian, (Ⅱ) enrichment or depletion in longevity family, and (Ⅲ) enrichment or depletion with aging. *p < 0.05; **p < 0.01; ***p < 0.001; ****p < 0.0001.

To investigate the characteristics of gut and oral microbiota in centenarians, we conducted 16S rRNA analysis across different cohorts. The alpha diversity analysis of the gut microbiota at ASV level in centenarians revealed that both the Chao richness index and the Shannon diversity index were significantly higher than those in the older and younger groups. However, there was no significant difference in the index of Shannon diversity and Pielou's evenness between centenarians and their lineal relatives. These results indicated that the richness of gut microbiota in centenarians is significantly higher than that in control groups, and the composition of bacteria is similar within the longevity family (Figure 3A). The alpha diversity analysis of oral microbiota showed that the lineal relatives of centenarians possessed the highest Chao richness index, while the centenarians' richness index was similar to the elderly and significantly higher than that observed in the young (Figure 3B). We hypothesized that the common loss of teeth among most centenarians might result in a decreased abundance of certain anaerobic bacteria in the alveolar region, which could subsequently impact microbial diversity compared to their lineal relatives.

Principal coordinate analysis (PCoA) of the gut microbiota composition revealed notable similarities in the microbiota composition between centenarians and their lineal relatives along the PCoA2 axis, distinct from the other two control groups. This suggests a unique microbiota profile shared in the longevity family, significantly different from the unrelated control group (Figure 3C). PCoA‐based beta diversity in the oral microbiota revealed a high overlap in the composition of oral microbiota between centenarians and their lineal relatives (Figure 3D). This may be attributed to the shared living environment and dietary habits within families, resulting in a similar oral microbiota environment.

The linear discriminant analysis effect size (LEfSe) analysis was further employed to identify significantly different genera to investigate the characteristic changes in gut and oral microbiota within the study cohort. In reference to the above analysis of serum metabolite, 3 categories of signature genera were identified: (Ⅰ) characteristic genera with centenarian signature, (Ⅱ) characteristic genera with longevity family signature, and (Ⅲ) genera of the aging signature (Figure 3E). Figure 3E illustrated the top 5 characteristic genera of gut microbiota and the top 3 characteristic genera of oral microbiota across all three signature categories, with the left side representing gut microbiota and the right side representing oral microbiota. These gut bacterial genera that met the above three characteristics were displayed in Figure S3. In the gut microbiota, a total of 16 characteristic genera were identified for centenarian signature, and the top 5 characteristic genera were consecutively listed as *Christensenellaceae_*R‐7_group, *Romboutsia*, *UCG‐002*, *Fusicatenibacter,* and *Anaerostipes*. Then, there were 9 characteristic genera with longevity family signature, with the top 5 genera being *Dorea*, *Phascolarctobacterium*, *Bacteroides*, *Erysipelotrichaceae_UCG‐003*, and *Eubacterium_hallii_group*. Furthermore, 6 characteristic genera represented the aging signature, with *Parabacteroides*, *Alistipes*, *Odoribacter*, *NK4A214_group,* and *Blautia* being the top 5 genera. Notably, the genus of *Christensenellaceae* and *Parabacteroides* have been reported to be closely associated with longevity in multiple studies of longevity population in China [17, 18], Italy [19], and Korea [20]. Similarly, these oral bacterial genera that met the above three characteristics were displayed in Figure S4. In the oral microbiota, 4 characteristic genera of centenarian signature were identified, with the top 3 being *Porphyromonas*, *Peptostreptococcus,* and *Eubacterium_nodatum_group*, all of which exhibited reduced abundance in centenarians. *Porphyromonas*, which has been reported to be associated with an increased risk of periodontitis, may have reduced abundance correlating with tooth loss in centenarians, leading to an overall decline in periodontal pathogenic bacteria. Additionally, 7 characteristic genera with longevity family signature were displayed in Figure S4, and in the top 3 genera, the abundance of *Neisseria* and *Haemophilus* showed a decrease in centenarians, whereas the abundance of *Achromobacter* increased. In the analysis of characteristic genera of aging signature, no genus varied significantly with age. Altogether, we identified characteristic genera from feces and saliva with altered abundances that correlated with longevity, including an increase of potential probiotics in gut microbiota and a reduction of harmful bacteria in oral microbiota, which highlighted the potential novel biomarkers and probiotic candidates for promoting healthy aging.

### Featured serum metabolites and gut/oral microbiota in centenarian compared to non‐centenarian group

**Figure 4:**
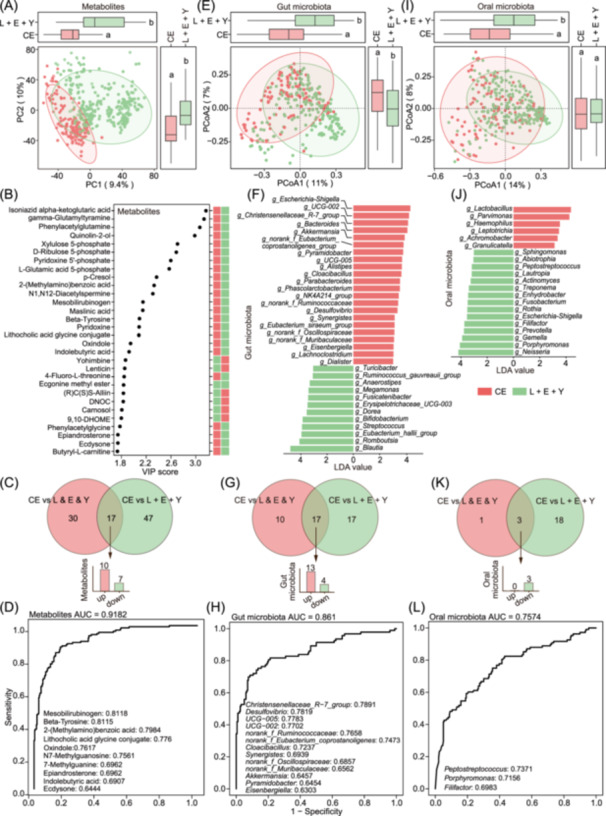
Characteristic serum metabolites and gut/oral microbiota in centenarian compared to non‐centenarian group. (A) PLS‐DA of serum metabolites between CE (*n* = 145) and non‐centenarians group (L + E + Y, *n* = 280). (B) The score plot of the variable importance in the projection (VIP) showed the top 30 serum metabolites driving the separation between group CE and group L + E + Y, with a cut‐off set at *p* < 0.05 and VIP > 1.5. (C) The Venn plot showed that 17 differential serum metabolites were shared between the centenarian signature metabolites depicted in Figure 2C and those mentioned above in (B). (D) Receiver operating characteristic (ROC) curve for logistic regression model to assess the separation efficacy of the 10 significantly upregulated metabolites identified in (C) for distinguishing CE from group L + E + Y. The ROC curve is produced by plotting sensitivity (true positive rate) on the *y*‐axis against 1–specificity (false positive rate) on the *x*‐axis. (E) PCoA plot based on Bray‐Curtis distances of gut microbiota in group CE and group L + E + Y. (F) Linear discriminant analysis Effect Size (LEfSe) to identify the differential gut genera between group CE and group L + E + Y (*p* < 0.05), with a cut‐off set at linear discriminant analysis (LDA) ≥ 3 and a relative abundance of >10% in the cohort. The 22 genera significantly increased, and 12 significantly decreased in CE. (G) The Venn plot showed that 17 differential gut genera were shared between the centenarian signature genera depicted in Figure 3E and those mentioned above in (F). Among them, 13 genera showed significant upregulation, and 4 showed downregulation in CE. (H) The separation efficacy of the 13 significantly upregulated gut genera identified from (G) was evaluated to distinguish CE from group L + E + Y. The *y*‐axis represented the sensitivity, and the *x*‐axis was 1–specificity. (I) PCoA based on Bray‐Curtis distances to analyze the oral microbiota in group CE and group L + E + Y. (J) LEfSe analysis to identify the differential oral genera between CE and group L + E + Y (*p* < 0.05), with a cut‐off set at LDA ≥ 3 and a relative abundance of >10% in the cohort. The 6 genera significantly increased, and 15 significantly decreased in CE. (K) The Venn plot showed that 3 differential oral genera were shared between the centenarian signature genera depicted in Figure 3E and those mentioned above in (J). (L) ROC curve for a logistic regression model to assess the separation efficacy of the three downregulated oral genera identified from (K) for distinguishing CE from group L + E + Y. The *y*‐axis represented the sensitivity, and the *x*‐axis was 1–specificity.

As mentioned above, centenarian have several specific serum metabolites and genera of gut and oral microbiota. To better elucidate the biomarkers of serum metabolites or gut/oral microbiota to uniquely differentiate the centenarian, we singled out the centenarian group (CE) for comparison with non‐centenarian populations (groups CE‐L + Elderly + Young) (Figure 4). Significant differences in serum metabolite profiles between CE and the non‐centenarians (CE‐L + Elderly + Young) were demonstrated in Figure 4A–C. For initial screening of differential serum metabolites, metabolites with a *p*‐value less than 0.05 and a value of variable importance in the projection (VIP) above 1.5 were selected, resulting in 64 differential serum metabolites [8]. PLS‐DA score plot was displayed in Figure 4A, exhibiting a significant difference in the centenarian group compared to the group L + E + Y in both PC1 and PC2 axes. The top 30 metabolites ranked by the VIP values are exhibited in Figure 4B. Notably, metabolites such as isoniazid alpha‐ketoglutaric acid, quinolin‐2‐ol, xylulose 5‐phosphate, and d‐ribulose 5‐phosphate are classified as organic acids, primarily involved in energy metabolic processes such as the tricarboxylic acid (TCA) cycle. Furthermore, by intersecting these 64 differential metabolites with the previously identified 47 centenarian‐specific metabolites (Table S3), we identified 17 shared differential serum metabolites (Figure 4C). Of these, 10 metabolites were significantly elevated in centenarians: 2‐(methylamino) benzoic acid, lithocholic acid glycine conjugate, epiandrosterone, ecdysone, N7‐methylguanosine, beta‐tyrosine, mesobilirubinogen, oxindole, indolebutyric acid, and 7‐methylguanine. To evaluate the discriminative potential of these mentioned 10 candidate biomarkers between centenarians and non‐centenarians (group CE‐L + Elderly + Young), receiver operating characteristic (ROC) analysis was performed. The area under the curve (AUC) for these metabolites' logistic regression reached 0.9182, indicating that the 10 metabolites mentioned above can be used as biomarkers for predicting longevity (Figure 4D).

To further elucidate the characteristics of the gut microbiota in centenarians, PCoA was employed to reveal significant differences in the gut microbial composition between centenarians and the non‐centenarian group (Figure 4E). Subsequently, differential genera of centenarians were identified by utilizing LEfSe (LDA ≥ 3) analysis and by considering relative abundance in the cohort (>10%). Several characteristic potential probiotic genera of centenarians overlapped with previous finding, as shown in Figure 3E, including *Christensenellaceae* R‐7 group, *Akkermansia*, *Pyramidobacter*, *Alistipes,* and *Parabacteroides*, suggesting that centenarians may possess a favorable gut microbiota composition (Figure 4F). The analysis of a Venn plot in gut microbiota was conducted to compare these 34 differential genera with the previously recognized 27 genera of centenarian signature. The analysis revealed 17 overlapping genera, among which 13 were significantly elevated in centenarians (Figure 4G). Then, ROC analysis was performed on these 13 gut genera, and the AUC reached 0.861, indicating that this analysis had good efficacy for predictive biomarkers of gut microbiota in distinguishing centenarians from the non‐centenarian group (Figure 4H).

Similarly, the PCoA analysis of the oral microbiota between centenarians and non‐centenarians revealed significant differences along the PCoA1 axis (Figure 4I). Differential oral genera of centenarians were identified by utilizing LEfSe (LDA ≥ 3) analysis and by considering relative abundance in the cohort (>10%). The analysis revealed a significant enrichment of *Lactobacillus* in the oral microbiota of centenarians, a well‐known probiotic, suggesting its potential role in maintaining oral health of centenarians (Figure 4J). Furthermore, the Venn plot of oral microbiota displayed the shared genera between these 4 differential genera and the previously recognized 21 genera with centenarian signature. The analysis revealed three overlapping genera, all of which exhibited a significant decrease in centenarians (Figure 4K). This reduction may be attributed to tooth loss in centenarians, as hypothesized in earlier discussions. Finally, ROC analysis of these three oral genera showed an AUC of 0.7574 (Figure 4L).

Overall, the analysis of serum metabolomics in conjunction with gut and oral microbiomes between centenarians and non‐centenarians has highlighted distinct serum metabolic and microbial traits characteristic of the centenarian population. These common differential metabolites and bacteria genera obtained by different grouping methods are worthy of further study. These screened metabolites and genera serve as potential biomarkers indicative of longevity and possess substantial predictive value.

### Healthy centenarian individuals harbored distinct serum metabolomic and gut/oral microbiota signatures

The metabolomic and gut microbiota profiles of elderly individuals have been reported to be closely linked to their health status in previous studies [21]. During our field investigations, significant individual variability in the physiological and cognitive functions of centenarians was also observed (Table S4). To assess the physical condition and cognitive abilities of centenarians, the frailty index (FI) was evaluated for each centenarian participant according to the comprehensive assessment that included the activity of daily living for assessing behavioral capacity and the minimum mental state examination for cognitive ability. In evaluation assessment system of FI, centenarian with a lower FI score indicates that he or she have better independence and cognitive function. Based on the FI scores, centenarians were further categorized into healthy centenarians (HC, FI < 11) and frail centenarians (FC, FI ≥ 11) to explore the characteristics of individuals with healthy longevity (Table S5).

**Figure 5:**
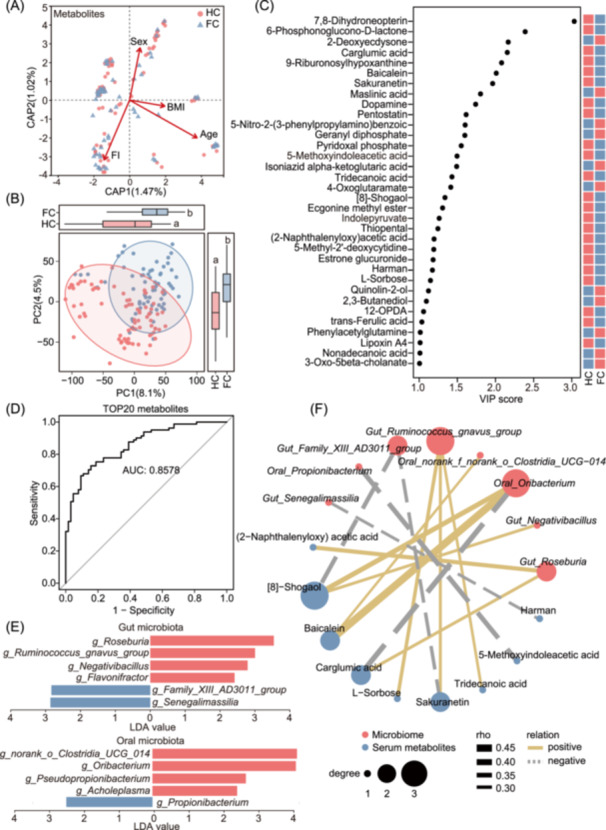
Healthy centenarian individuals harbor distinct metabolomic and gut/oral microbiota signatures. (A) Distance‐based Redundancy Analysis (dbRDA) of serum metabolites and clinical indicators in healthy centenarians (HC, *n* = 81) and frail centenarians (FC, *n* = 64). (B) PLS‐DA of serum metabolites between HC and FC. (C) The VIP score plot showed the total 34 differential serum metabolites between HC and FC groups, with a cut‐off set at VIP > 1 and *p* < 0.05. (D) ROC curve to evaluate the classifying ability of the top 20 metabolites in distinguishing between the HC and the FC group. The *y*‐axis represented the sensitivity, and the *x*‐axis was 1–specificity. (E) LEfSe analysis to identify the differential genera in gut and oral microbiota between HC and FC group (LDA ≥ 2), with a cut‐off set at a relative abundance of >10% in the cohort and *p* < 0.05. (F) Spearman's rank correlation for analyzing the relationship between serum metabolites and gut/oral microbiota that are elevated in HC.

The distance‐based redundancy analysis (db‐RDA) was employed to evaluate the effects of age, gender, BMI, and FI on metabolomic profiles in CE, and the FI was observed to be the primary determinant influencing the distribution of serum metabolites (Figure 5A). Then, PLS‐DA was used to display the variance in metabolites between HC and FC, revealing significant differences in metabolite distribution between healthy centenarians and frail centenarians (Figure 5B). A total of 34 serum metabolites which were differentially expressed in HC were screened out, with criteria of VIP > 1 and *p* < 0.05 (Figure 5C). Among the characteristic serum metabolites of HC, 6‐phosphoglucono‐D‐lactone and Dopamine have attracted our attention. The 6‐phosphoglucono‐D‐lactone is known as an essential intermediate in the hexose monophosphate pathway, in which process NADPH is concomitantly generated — a critical component of the cellular antioxidant defense system — to protect cells from oxidative damage [22]. Dopamine, an important neurotransmitter, has well‐established roles in cognitive function and emotional regulation, as validated by numerous studies [23, 24]. Both mentioned metabolites have the ability to help centenarians maintain physical vitality and mental well‐being. In addition, various food‐source substances were enriched in HC, such as 8‐gingerol, baicalein, and sakuranetin [25]. The 8‐gingerol and baicalein are the anti‐inflammatory compounds derived from herbal medicine, whereas sakuranetin, a flavonoid compound, has been confirmed to exert protective effects on rat brain cells through antioxidant mechanisms. This indicates that healthy centenarians may maintain good physical condition through their dietary intake. Furthermore, the ROC curves were constructed using the top 20 metabolites ranked by VIP scores to assess their discriminative validity between HC and FC, and the AUC of the ROC curve reached 0.8578 (Figure 5D). Altogether, serum metabolites serve as a clear indicator for distinguishing the health status and frailty levels of centenarians. Moreover, individuals who achieve healthy longevity often demonstrate superior antioxidant capabilities and emotional regulation abilities.

Similarly, LEfSe analysis was employed to analyze the characteristics of the gut and oral microbiota in HC (Figure 5E). In the gut microbiota, the HC group showed an enrichment of genera such as genera *Roseburia*, *Ruminococcus gnavus group*, *Negativibacillus,* and *Flavonifractor*, along with a reduction in *Senegalimassilia* and *Family XIII AD3011 group*. Among them, the genus *Roseburia* is known as the cornerstone genus of the gut microbiota and a common producer of short‐chain fatty acids. The genus *Flavonifractor* contains the well‐known probiotic *Flavonifractor plautii* which is famous for its ability to reduce the inflammatory response in adipose tissue [26] and acute colitis [27], and to prevent arterial stiffness [28]. Whereas, in the oral microbiota, the HC group demonstrated an enrichment of the genera *Acholeplasma*, *Pseudopropionibacterium*, *Oribacterium*, and *g_norank_o_Clostridia_UCG_014*, along with a decrease in *Propionibacterium*. Previously, *Oribacterium* has been demonstrated to be a beneficial oral genus capable of significantly reducing the risk of plaque and gingival bleeding [29, 30]. The best‐known species of the genus *Propionibacterium* is *Propionibacterium acidifaciens*, an oral pathogen related to dentinal caries lesions [31]. A reduction of *Propionibacterium* suggests that healthy centenarians have a lower risk of suffering from oral diseases.

Finally, the correlation network was used to elucidate the relationships among these elevated serum metabolites and differential genera of gut and oral microbiota in HC (Figure 5F). Plant‐derived sakuranetin was found to be positively correlated with the gut genus *Ruminococcus gnavus group*, which implied that the gut microbiota was involved in sakuranetin metabolism. Additionally, food‐source metabolites, 8‐gingerol and baicalein were significantly positively correlated with the oral genus *Oribacterium*, suggesting that dietary intake of these substances may contribute to maintaining oral health by raising the levels of *Oribacterium*. Overall, within the centenarian between healthy and frail individuals, significant differences in metabolite profiles and microbiota compositions were observed and were associated with diet.

### Tryptophan metabolism shaped the link between the serum metabolomic and microbiota from gut and oral cavity

**Figure 6:**
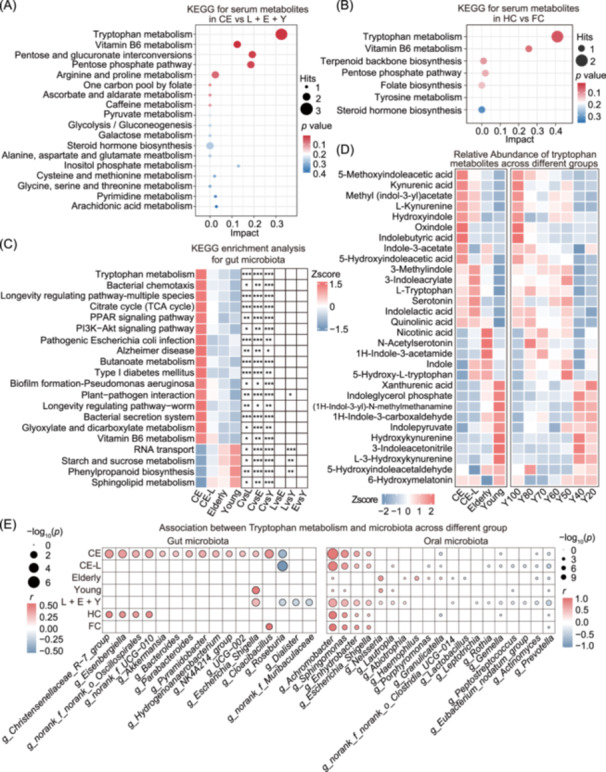
Tryptophan metabolism was enriched from the conjoint analysis of metabolomic and gut/oral microbiota. (A) KEGG enrichment analysis for differential serum metabolites between CE and non‐centenarians (CE‐L + Elderly + Young). A total of 18 pathways were identified, with the tryptophan metabolism pathway being the most significant. (B) KEGG enrichment analysis derived from differential serum metabolites in the comparison of HC and FC. The tryptophan metabolism pathway was the most significant in seven identified KEGG pathways. (C) KEGG enrichment analysis based on 16S rRNA sequencing of the gut microbiome, revealing a significant enrichment in the tryptophan metabolism pathway in the CE group. *p < 0.05; **p < 0.01; ***p < 0.001. (D) The abundance of all tryptophan‐related metabolites in serum untargeted metabolomics across different groups, including CE, CE‐L, Elderly, and Young, as well as age‐based groups ranging from Y20 to Y100 regardless of kinship. There are nine tryptophan‐related metabolites exhibiting high abundance in both the CE and Y100 group (≥100 yrs). (E) Spearman's rank correlation analysis between the abundance of tryptophan metabolic pathways and the abundance of differential gut microbiota (left) as well as oral microbiota (right) in different groups.

To gain a deeper understanding of the functions of serum metabolites and microbiota in centenarians, the enrichment of KEGG pathways was further analyzed. The significantly differential serum metabolites in CE were found to be enriched in 18 KEGG pathways, with the top 5 enriched pathways including tryptophan metabolism, vitamin B6 metabolism, interconversion of pentoses and glucuronates, the pentose phosphate pathway, and arginine and proline metabolism (Figure 6A). Likewise, we performed KEGG enrichment analysis on the differential serum metabolites between HC and FC groups. The 7 metabolic pathways were involved, with the top 3 being tryptophan metabolism, vitamin B6 metabolism, and terpenoid backbone biosynthesis (Figure 6B). Furthermore, functional prediction analysis by PICRUSt2 from 16S rRNA sequencing data of gut and oral microbiota was conducted separately. According to the centenarian signature, as mentioned above in Figure 2, the tryptophan metabolism pathway exhibited highest fold change compared to the other three groups in the top 20 enriched pathways (Figure 6C). Our results illustrated the potential importance of tryptophan as a link between serum metabolism and gut microbiota for maintaining health during the aging process.

Given the importance of the tryptophan metabolic pathway in serum metabolites and gut microbiota, we compared the relative abundances of all 30 tryptophan‐related metabolites in the serum metabolome (Figure 6D). To better understand the trends with age of tryptophan‐related metabolites, all individuals were reassigned into the age gradient group, ensuring that each cohort had no fewer than 20 individuals, and named as Y20 (20–29 yrs), Y40 (40–49 yrs), Y50 (50–59 yrs), Y60 (60–69 yrs), Y70 (70–79 yrs), Y80 (80–89 yrs), and Y100 groups (≥100 yrs), respectively. 9 metabolites were found to exhibit not only the highest abundance in the CE group but also the highest prevalence in Y100 group. These metabolites include 5‐MIAA, kynurenic acid, methyl (indol‐3‐yl) acetate, L‐kynurenine, hydroxyindole, oxindole, indolebutyric acid, indole‐3‐acetate and 5‐hydroxyindoleacetic acid. As previously described in Figure 2C, we found that oxindole and indolebutyric acid were significantly enriched in the CE group, while 5‐MIAA was notably higher in the HC group, as shown in Figure 5C. Additionally, seven metabolites appeared similar abundance between CE and CE‐L group, and further analysis showed that three metabolites have no significant difference between CE and CE‐L, but there is a significant difference in CE compared to Elderly and Young, specifically for serotonin, indolelactic acid and 6‐hydroxymelatonin (Figure S5). Serotonin [32], a well‐known neurotransmitter, plays a critical role in regulating mood and cognitive function, and its elevation can promote the breakdown of fat in adipocytes, thereby aiding in maintaining blood sugar levels and controlling obesity [33]. Indolelactic acid [34] is noted for its anti‐inflammatory and potential antiviral activities, indicating that these metabolites may contribute to health and longevity. The 6‐Hydroxymelatonin is a primary metabolic of melatonin, metabolized by CYP1A2, which has been reported [35] to protect against oxidative stress and iron‐induced neurotoxicity [36]. The mentioned metabolites of serotonin, indolelactic acid, and 6‐hydroxymelatonin exhibit distinct enrichment characteristics within longevity families, suggesting that the metabolism of these longevity families is more beneficial for maintaining health and promoting longevity.

Moreover, Spearman's rank correlation was employed to analyze the involvement of gut and oral microbiota in tryptophan metabolism pathway across different groups (CE, CE‐L, Elderly, Young, non‐centenarians, HC, and FC). It was observed that four genera in the gut microbiota were positively associated with the tryptophan pathway in both the CE and HC groups, including *Christensenellaceae* R‐7 group, *Eisenbergiella*, *norank_f_norank_o_Oscillospirales* and *norank_f_UCG‐010*. Meanwhile, we found seven oral genera significantly were positively correlated with the tryptophan pathway in both the CE and HC groups, including *Achromobacter*, *Sphingomonas*, *Enhydrobacter,* and *Escherichia Shigella* (Figure 6E).

Following these findings on the serum metabolites and microbiota related to the tryptophan pathway, we next focus on exploring the interconnections among tryptophan metabolites, gut and oral microbiota to identify the key metabolites.

### Key metabolite in tryptophan metabolism revealed by integrative analysis and its potential function

**Figure 7:**
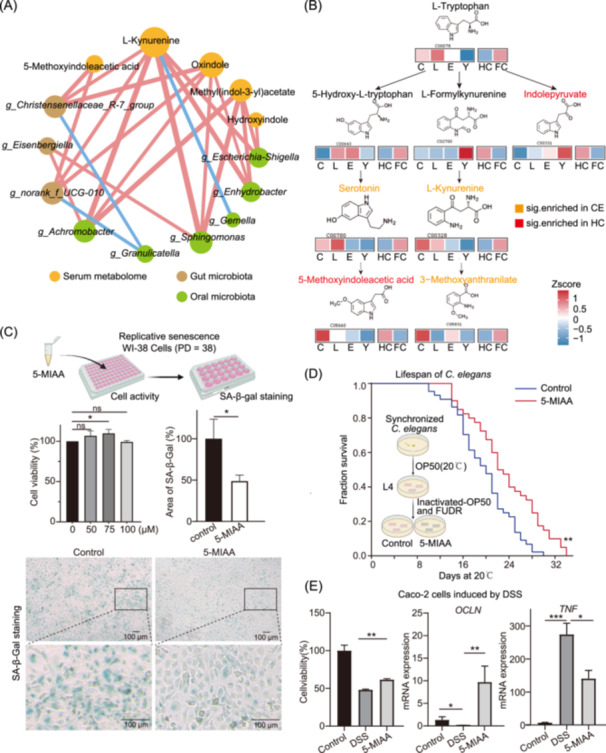
Identification and validation of 5‐Methoxyindoleacetic acid (5‐MIAA) as a key serum metabolite in the tryptophan pathway. (A) Correlation network plot illustrating the relationships between tryptophan‐related metabolites and significant differential genera of the gut and oral microbiota, with *r* ≥ 0.2 and *p* < 0.05. (B) Simplified schematic diagram of tryptophan pathway, presenting the significantly different tryptophan‐related metabolites, with Z‐score heatmaps representing their abundance in the CE, CE‐L, Elderly, Young, as well as HC and FC groups. Molecular formulas and IDs of the metabolites were obtained from the KEGG database (http://www.genome.jp/kegg/). (C) Effect of 5‐MIAA on the replicative senescence of WI‐38 cells (PD = 38). The CCK‐8 assay was used to assess cell viability at 5‐MIAA concentrations of 50, 75, and 100 μM (left). SA‐β‐Gal staining was performed for WI‐38 cells treated with 100 μM 5‐MIAA (right). (D) Lifespan curves of wild‐type *Caenorhabditis elegans* maintained at 20°C on NGM plates supplemented with or without 1 mM 5‐MIAA. Statistical significance was determined using log‐rank (Mantel–Cox) test. (E) Anti‐inflammatory effects of 5‐MIAA on dextran sulfate sodium salt (DSS)‐induced Caco‐2 cells. The CCK‐8 assay of 5‐MIAA at the concentrations 100 μM (left) and the mRNA expression levels of *OCLN* and *TNF* in DSS‐induced Caco‐2 cells cocultured with 5‐MIAA. Control: the cells cultured without DSS; DSS: the cells cultured with DSS for inflammation modeling; 5‐MIAA: the DSS‐induced cells co‐treated with 5‐MIAA. Compared to the model group, the alleviation of inflammation was observed in the co‐treatment with 5‐MIAA. *p < 0.05, **p < 0.01, ***p < 0.001.

To identify the key metabolites of tryptophan pathway that promote healthy longevity, correlation network and pathway reconstruction were further conducted to analyze. Firstly, we performed a correlation analysis among nine tryptophan metabolites enriched in both CE and Y100 group, four genera in gut microbiota, and seven genera in oral microbiota. Significant positive correlations were observed between genus *Christensenellaceae* R‐7 group and 5‐MIAA (*r* = 0.37, *p* = 5.62E‐08), L‐Kynurenine (*r* = 0.27, *p* = 9.20E‐05), and oxindole (*r* = 0.39, *p* = 5.32E‐09), respectively (Figure 7A). The genus *Christensenellaceae* R‐7 group has been previously reported as a potential probiotic, and these findings suggest that it may participate in the tryptophan metabolism by influencing the level of 5‐MIAA, L‐Kynurenine, and oxindole. We attempted to recover the identified tryptophan metabolites to the tryptophan pathway and found that 19 of 30 metabolites were present in the tryptophan pathway. Among them, 5 serum metabolites, such as 5‐MIAA, 3‐Methoxyanthranilate, serotonin, L‐Kynurenine and indolepyruvate, showed significant variation in either the CE or HC group, which were marked in Figure 7B, along with their upstream metabolites: 5‐Hydroxy‐L‐tryptophan, L‐Formylkynurenine and L‐Tryptophan. 5‐MIAA was found to be the singular metabolite which was not only enriched in the CE group but also in the HC group (Figure 7B). 5‐MIAA is a downstream product of L‐tryptophan metabolism, and its increase may be attributed to the elevated levels of its precursor — serotonin, a neurotransmitter known for its various beneficial effects. Studies have reported that plasma 5‐MIAA levels in healthy individuals, determined using UHPLC‐MS/MS, range from 118 to 578 pM [37]. It is speculated that 5‐MIAA is not only an important gut microbiota‐derived tryptophan metabolite but may also have significant biological functions in promoting healthy aging.

Therefore, the potential of 5‐MIAA for life extension was further validated through in vitro and in vivo experiments. First, WI‐38 cells, a well‐known model of replicative senescence, were used to assess whether 5‐MIAA could delay senescence. The effects of different 5‐MIAA concentrations on WI‐38 cell viability (PD = 38) were examined to determine the maximum tolerance concentration. The CCK‐8 assay showed that within a range of 0–1000 μM, 5‐MIAA was nontoxic at concentrations below 100 μM. Additionally, compared to 24 h, the cell viability at 72 h of treatment increased by 52.30%, 15.97%, and 6.70% at 5‐MIAA concentrations with 50, 75, and 100 μM, respectively. Notably, WI‐38 cells exhibited sustained proliferation after 72 h of treatment with 50–100 μM 5‐MIAA (Figure 7C, Figure S6). Subsequently, WI‐38 cells cultured with 100 μM of 5‐MIAA for 72 h were subjected to senescence‐associated β‐galactosidase (SA‐β‐Gal) staining. Compared to controls, these cells displayed significantly reduced staining, with the average integrated density decreasing by 51.23% (*p* = 0.023), suggesting that 5‐MIAA may delay senescence (Figure 7C).

To further validate these findings in vivo, lifespan assays were conducted using *Caenorhabditis elegans* (*C. elegans*). Animals treated with 0.5% DMSO had a mean lifespan of 19.95 ± 0.74 days, whereas those treated by 1 mM 5‐MIAA had a mean lifespan of 22.93 ± 0.96 days (Figure 7D). The average lifespan of *C. elegans* was extended by 14.94% (*p* = 0.003), indicating that 5‐MIAA promotes life extension.

Given the strong association between 5‐MIAA and gut microbiota, we evaluated its effects on intestinal mucosal cells (Caco‐2 cell) to further elucidate its mechanism in the intestinal epithelium. To assess the protective potential of 5‐MIAA, we first determined its impact on Caco‐2 cell viability under dextran sulfate sodium (DSS)‐induced stress. The half‐maximal inhibitory concentration (IC50) of DSS for Caco‐2 cells was 60 mg/mL, while the maximum safe dose of 5‐MIAA was 100 μM (Figure S7). Subsequently, Caco‐2 cells were co‐stimulated with DSS and 5‐MIAA to evaluate the changes in cell viability (Figure 7E). The results showed that cell viability in the 5‐MIAA‐treated group was significantly higher than that in the DSS‐model group (*p* = 1.96E‐04), suggesting that 5‐MIAA effectively protects Caco‐2 cells from DSS‐induced viability loss. Additionally, we examined the mRNA expression levels of tight junction‐associated proteins and inflammatory cytokines in DSS‐treated Caco‐2 cells co‐stimulated with 5‐MIAA (Figure 7E). Compared to the model group, co‐treatment with 5‐MIAA significantly increased *OCLN* mRNA levels (*p* = 0.0094), while reducing the expression of the inflammatory cytokine *TNF* (*p* = 0.0215). These findings indicated that 5‐MIAA effectively mitigates DSS‐induced damage in Caco‐2 cells, enhancing the barrier‐protective function of intestinal epithelial cells.

Collectively, we have identified 5‐MIAA, a key tryptophan metabolite, as strongly correlated with genus *Christensenellaceae* R‐7 group in centenarians, particularly healthy individuals. It plays a pivotal role in delaying cell senescence, extending lifespan, and mitigating inflammation in intestinal cells.

## DISCUSSION

In this study, we revealed distinct variation characteristics in numerous metabolites and microbiota exhibited between centenarians and the control group. These include metabolites and microbes associated with the centenarian signature which may influences on longevity, the longevity family signature which suggests a genetic predisposition, and the aging signature which associated with the biological process of aging. Consistent with previous studies, our findings illustrated that centenarians underwent a metabolic remodeling distinct from other aging populations, and the diversity of the gut microbiota in centenarians was significantly increased, sharing a similar composition with their lineal relatives [9, 13]. Certain serum metabolites, as well as gut and oral microbiota, are all enriched in the tryptophan metabolism, indicating their interconnection and mutual influence. Furthermore, the abundance of key serum metabolites in tryptophan metabolism — 5‐MIAA was elevated in centenarians and even in healthy centenarians. The 5‐MIAA was proven to exert effects that delay senescence and promote longevity with in‐vitro and in‐vivo experiments, and the *Christensenellaceae* R‐7 group may be directly or indirectly involved in the metabolic process of 5‐MIAA, thereby maintaining individual health. All the above results indicated that tryptophan‐related metabolites may exert the potential to promote lifespan extension.

Through comprehensive analysis of blood biomarkers, several changes in indices, such as lower BMI and ALB, were observed in centenarians. The lower BMI in centenarians may reflect reduced fat and muscle mass due to age‐related sarcopenia, as well as metabolic adaptations linked to longevity, such as caloric restriction. Similarly, reduced albumin levels could suggest mild age‐related changes in liver function or nutritional status. These altered health indices may represent a unique combination of physiological adaptations and protective mechanisms that contribute to their exceptional longevity. As previously described, centenarians have reached the limits of human lifespan. They are referred to as “centenarians” or “longevity individuals” primarily because they largely evade, delay, or avoid age‐related diseases.

In our earlier review of age‐related and longevity‐associated metabolomic profiles, we summarized that amino acids, fatty acids, and bile acids are among the key metabolites that often change with age [38]. Regarding serum metabolomics and gut microbiome changing with age, we found that L‐tyrosine, L‐methionine S‐oxide, and glycocholic acid exhibited age‐related increases in our study, consistent with findings from previous studies [5, 39]. Additionally, the gut microbiota of aging is often associated with a decline in beneficial bacteria, such as *Bifidobacterium*, and an increase in potentially harmful bacteria, including *Clostridium* and *Klebsiella pneumoniae* [40, 41]. However, we observed an age‐related enrichment of *Odoribacter* in our study, which has been previously reported as a signature taxa in centenarians and play beneficial roles in host health, such as modulating inflammation [9, 42].

Through an comprehensive analysis of serum metabolomics, gut and oral microbiome, several potential probiotic genera were identified with high abundance in the gut of centenarians, such as *Akkermansia*, *Alistipes*, and *Christensenellaceae* R‐7 group. More precisely, genus *Alistipes* has been reported to exhibit dual roles: it offers protective effects in conditions like colitis, autism spectrum disorders, and various liver and cardiovascular fibrotic diseases, while also showing pathogenicity in anxiety, myalgic encephalomyelitis/chronic fatigue syndrome, depression, pervasive developmental disorder not otherwise specified and colorectal cancer [43, 44, 45, 46, 47]. Additionally, *Akkermansia muciniphila* has been shown to improve metabolism, alleviate cognitive impairment, exert antitumor effects, and enhance gut barrier function [48, 49, 50, 51]. The *Christensenellaceae* has been reported to regulate liver lipid metabolism by inhibiting fat synthesis, preventing obesity, and modulating key antiaging indicators such as blood glucose and leptin [52]. Overall, the increased abundance of various potential probiotics in the gut of centenarians contributes to their ability to maintain better “healthy aging”.

In the realm of oral microbiomics, although the oral microbiota composition of centenarians is less complex than that of the gut microbiota, it exhibits unique α and β diversity, particularly showing similarities with their lineal relatives. This characteristic is primarily influenced by factors such as tooth loss, living environment, and dietary habits. Notably, in the centenarian group, there is a significant decrease in the abundance of *Porphyromonas* and a marked increase in *Lactobacillus*. The reduction in *Porphyromonas* may be associated with decreased periodontal pathogens due to tooth loss, thereby affecting tryptophan metabolism. Studies have shown that strain *Porphyromonas* ATCC33277 can lead to decreased serum tryptophan metabolites in mice, accompanied by increased fat mass and impaired glucose tolerance, further indicating that tryptophan metabolic defects may be a significant factor in *Porphyromonas*‐induced metabolic disorders [53]. On the other hand, *Lactobacillus*, commonly recognized as a gut probiotic [54, 55, 56], is also found in high abundance in the oral microbiota of centenarians, which is not surprising given the shared pathway between the gut and oral cavity. Our study also found a strong positive correlation between serum metabolites such as baicalin and 8‐gingerol and the beneficial oral bacterium *Oribacterium* in healthy centenarians. Overall, despite the reduced diversity in the oral microbiota of centenarians, the decrease in pathogenic bacteria and increase in beneficial bacteria, along with the potential involvement of the microbiota in the biotransformation of metabolites, contribute to maintaining the homeostasis of the oral microbiota in centenarians.

Our study found that the tryptophan pathway was the primary enriched metabolic pathway in centenarians, especially in healthy centenarians, suggesting that the tryptophan pathway may be an important mechanism to achieve “healthy aging.” Previous studies have shown that pathways such as TCA cycle, methionine metabolism, and arginine metabolism also showed better network integrity and metabolic regulation in the longevity [5, 57]. The tryptophan metabolic pathway has also been documented to promote intestinal and systemic homeostasis in health and disease by exerting anti‐inflammatory and antioxidant effects [58, 59]. Our study conveyed that numerous tryptophan metabolites are significantly enriched in centenarian and even healthy centenarians. The metabolites and gut and oral microbiota associated with the tryptophan pathway were further explored in depth. The genus *Christensenellaceae* R‐7 group in the gut microbiota was negatively correlated with genus *Granulicatella* in the oral microbiota, and the latter is known to be a common bacterium in the human oral cavity and is associated with periodontal disease [60, 61]. Furthermore, A strong positive correlation was found between *Christensenellaceae* R‐7 group and 5‐MIAA, indicating that 5‐MIAA was closely associated with the gut microbiota. Previously, 5‐MIAA has been recognized as an nuclear factor erythroid 2‐related factor 2 (Nrf2) activator generated by gut microbiota [62]. Activation of Nrf2 typically influences the expression of genes related to intestinal epithelial barrier function and immune responses, which is crucial for maintaining gut homeostasis [63]. So far, two studies have been reported the function of 5‐MIAA association with gut microbiota. It has been shown that 5‐MIAA, produced by *Lactobacillus rhamnosus*, activates the Nrf2 antioxidant response in the liver, protecting against oxidative liver injury [62]. Additionally, 5‐MIAA improves oxidative stress in high‐fat diet‐induced alcoholic fatty liver mice and is significantly positively correlated with *Lactobacillus* [64]. As a metabolite of serotonin, 5‐MIAA is involved in the regulation of biological rhythms and sleep, suggesting its potential role in regulating circadian rhythms and aging [65]. These evidence further supported the potential beneficial effects of 5‐MIAA on host health and highlight its promise as a prospective antiaging metabolite.

Several limitations of this study should be discussed. Firstly, the serum metabolome may be influenced by multiple factors such as life schedule, exercise frequency, and dietary habits. Our study was based on family surveys and geographic sampling, ensuring similarity and homogeneity in participants' dietary habits, but dietary structures, and physical activity, age‐related degenerative changes in the digestive system may lead to shifts in dietary preferences and metabolic capacity, further affecting gut microbiota and metabolic products. Secondly, we characterized the gut and oral microbiota using 16S rRNA sequencing, providing limited information for phylogenetic analysis of microorganisms. Integrating metagenomic analysis could further elucidate the relationship between microbiota and tryptophan metabolites, aiding in a deeper understanding of microbial metabolism mechanisms.

## CONCLUSION

In conclusion, our study identified the characteristic profiles of the serum metabolome, gut, and oral microbiota in centenarian individuals and longevity families, as well as available trails with aging. Serum metabolites, as well as gut microbiota, are promising biomarkers of longevity and healthy aging. Additionally, our study further reveals that the tryptophan pathway may be an important mechanism for individuals to achieve healthy aging. We have unearthed the key tryptophan metabolite 5‐MIAA, which was found to exhibit effects of delaying cellular senescence and promoting lifespan. Overall, this study will provide new scientific insights and directions for achieving healthy longevity.

## METHODS

### Volunteers recruitment and sample collection

The cohort for this study was recruited from Suixi county, Zhanjiang city, located in the western region of Guangdong province, China, which has been elected as the “International Longevity and Health Care Base.” A total of 425 participants were enrolled, including 145 centenarians, as well as 126 lineal relatives of the centenarians, 92 elderly individuals and 62 young individuals.

All participants underwent medical history interviews, questionnaire surveys, and routine physical examinations. Participants were included in this study when met these criteria: (1) aged ≥ 20; (2) absence of major diseases, such as cancer, autoimmune diseases, or Alzheimer's disease; and (3) no history of using hormones, immunosuppressants, or anticoagulant medications. In addition, the elderly and young control group have been investigated the history of family longevity, and participants were recruited when he/she has no blood relatives over 80 years old within three generations. Exclusion criteria for all participants were as follows: (1) cognitive impairment, severe organic diseases, or history of major trauma or surgery within the past 2 years; (2) history of *Clostridium difficile* infection or use of medications that may affect gut microbiota (e.g., antibiotics or hormones) within the 3 months before sample collection; (3) use of antimicrobial agents or anti‐inflammatory drugs (such as NSAIDs) within the past 3 months; and (4) a history of gastrointestinal diseases, including gastrointestinal cancer, chronic or infectious gastroenteritis, colitis, gastritis, gastrointestinal ulcers, or gastrointestinal bleeding. For the control group, additional exclusion criteria included a history of gastrointestinal diseases, diabetes, hypertension, hyperlipidemia, coronary heart disease, cerebrovascular disease, autoimmune diseases, inflammatory bowel disease, severe organ damage, or systemic diseases.

Participants were divided into four groups: the centenarian group (CE), the lineal relatives of centenarians group (CE‐L), the elderly control group (Elderly), and the young control group (Young). Blood, fecal, and saliva samples were collected from volunteers who were willingness to provide them, resulting in a total of 425 serum samples, 347 saliva samples, and 291 fecal samples. Blood and fecal samples were sent to the Clinical Laboratory of Affiliated Hospital of Guangdong Medical University for biochemical analysis and routine fecal testing. Additionally, a frailty index (FI) was calculated based on medical history and questionnaire results for the centenarian group. Centenarians were further divided into a healthy group and a frail group based on the top half of FI scores. Detailed information on the frailty index is provided in Table S4.

### Untarget serum metabolism

Fasting venous blood samples were collected from participants and then left to clot at room temperature for 30 min. The serum was obtained after being centrifuged at 3500 rpm for 10 min and frozen at −80°C until use for untargeted metabolomics analysis by liquid chromatograph mass spectrometer (LC‐MS). In brief, the serum samples were mixed with methanol, vortexed and centrifuged at 12,000 rpm for 10 min at 4°C. Then, the supernatant was collected, concentrated and dried for detection.

### 16S rRNA sequencing for gut and oral microbiota

The FastPure DNA Extraction Kit (TransGen Biotech, Beijing, China) was used to extract the microbial genomic DNA from fecal and saliva samples, according to the manufacturer's instructions. Then, the V3‐V4 regions of 16S rRNA gene of the bacteria were amplified through the primer pair 338F (5′‐ACTCCTACGGGAGGCAGCAG‐3') and 806R (5′‐GGACTACHVGGGTWTCTAAT‐3′) on a T100 Thermal Cycler (BIO‐RAD, USA). The amplification for PCR was conducted under the following processes: initial denaturation at 95°C for 3 min, followed by 27 cycles of 95°C for 30 s, 55°C for 30 s, and 72°C for 45 s, with a final extension at 72°C for 10 min, and held at 4°C. The PCR products were sequenced on Illumina NextSeq 2000 PE300 platform at Majorbio Bio‐Pharm Technology Co., Ltd.

### Data analysis

Metabolomics analysis: the raw data was converted into the mzXML format by ProteoWizard (v3.0.8789), and then the XCMS (R package, v3.1.3) was used to identify, filtrate and align the peaks. The metabolites identified by accuracy mass (<30 ppm) and MS/MS data were matched with HMDB (https://www.hmdb.ca/), massbank (https://massbank.jp/), LipidMaps (http://www.lipidmaps.org), mzcloud (https://www.mzcloud.org) and KEGG (http://www.genome.jp/kegg/). After scaling data, models were established on partial least‐square discriminant analysis (PLS‐DA). Moreover, all the models were evaluated by overfitting with methods of permutation tests. The discriminating metabolites were determinated by the VIP. The *p*‐value, VIP produced by PLS‐DA, fold change (FC) was applied to discover the contributable‐variable.

16S rRNA sequencing: after quality control, the raw paired‐end sequences were performed using the fastp software (https://github.com/OpenGene/fastp, v0.19.6), and paired reads were merged using FLASH (http://www.cbcb.umd.edu/software/flash, v1.2.11). Processed sequences were denoised using DADA2 (or Deblur) plugins in the Qiime2 pipeline under default parameters, resulting in amplicon sequence variants (ASVs). Taxonomic classification of ASVs was conducted with the Naive Bayes (or Vsearch or Blast) classifier in Qiime2, based on the SILVA 16S rRNA gene database (v138). Functional predictions of microbial communities were performed through PICRUSt2 software (v2.2.0).

### The ability of alleviating cellular senescence in human embryonic lung cells WI‐38

CCK8 assay: the cell viability of WI‐38 was tested by the CCK8 method. WI‐38 cells (Abbkine, Wuhan, China) at a concentration of 5.0 × 10^3^ cells per 100 μL of MEM medium (Gibco, USA) were added to a 96‐well plate. The assay was conducted using a commercial kit from Beyotime Biotechnology (C0038, Shanghai, China). A solution of 10 μL CCK8 was infused into each well after culturing with different concentrations of 5‐MIAA. The absorbance at 450 nm was measured by MicroplateReader (ThermoFisher, USA). Cell viability was calculated as: Cell viability (%) = [A (metabolite) − A (blank)]/[A (control) − A (blank)] × 100%.

Cellular Senescence‐associated β‐galactosidase (SA‐β‐gal) staining: WI‐38 cells were cultured in MEM medium to reach replicative senescence (PD = 38). SA‐β‐gal staining was conducted using the commercial kit (No.C0602, Beyotime, Shanghai, China). The cells were treated by MEM medium with and without 100 μM 5‐MIAA, respectively. After incubation for 72 h, the WI‐38 was washed one time with PBS and fixed with the fixative solution for 15 min at room temperature. Subsequently, the cells were stained according to the kit's manual and incubated at 35°C without CO_2_ overnight. The cells were washed three times with PBS for 3 min each and then photographed using the MF52‐M microscope (Mshot). Pictures were taken for over three visions per well, and the ImageJ software was used to calculate the percentage of cells staining positively with SA‐β‐gal.

### The lifespan measurement of metabolites by *Caenorhabditis elegans*

The strain and maintenance of *Caenorhabditis elegans* (*C. elegans*): the wild‐type N2 strain of *C. elegans* (SunyBiotech, Fuzhou, China) was used. All *C. elegans* were cultivated on the standard plates of nematode growth medium (NGM) at 20°C with *Escherichia coli* OP50 as a food source.

Lifespan assay: Synchronized L4 larvae of worms were transferred to three plates of NGM (nearly 30 per plate, diameter 3.5 cm) containing inactivated OP50 and 20 μM of FUDR (F0503, Sigma, Germany). Then, worms were fed with 5‐MIAA in 0.5% DMSO at concentrations of 0.05, 0.1, 0.5, 1, 2, and 10 mM, with 0.5% DMSO in M9 buffer as the control. The day of the transfer to the plate was recorded as Day 0. To ensure the potency of 5‐MIAA and sufficient food, worms were transferred to fresh plates every other day. The survival data were plotted with the Kaplan–Meier curves in GraphPad Prism (v10). Statistical significance was tested by the log‐rank (Mantel–Cox) method.

### The anti‐inflammatory ability in DSS induced Caco‐2 cells

Caco‐2 cells were cultured in DMEM (Gibco, USA) with 10% FBS (NZ500, NEWZERUM), 1% NEAA (Gibco, USA), and 0.5% penicillin‐streptomycin (Gibco, USA). Upon reaching 85% confluence, they were digested with 0.25% trypsin (Gibco, USA), planted in 96‐well plates at 6000 cells per well, and incubated for 24 h. After PBS washing, metabolites were added at final concentrations of 10, 100, 250, 500, and 1000 μM, and cells were incubated for 24 and 48 h. Then, 100 μL of 10% CCK‐8 solution was added, and the OD value at 450 nm was measured after 2 h.

DSS (60316ES25, Yeasen Biotechnology, Shanghai, China) was suspended in sterile water, filtered through a 0.22 μm membrane, and diluted in culture medium to various concentrations (0–100 mg/mL). Cell viability was assessed using the CCK‐8 after treating cells in 96‐well plates with 60 mg/mL DSS and different metabolites for 24 or 48 h. Total RNA was extracted using the Trizol (Invitrogen, USA) method, and cDNA was synthesized with PrimeScript™ RT Master Mix (RR036A, Takara, Japan). RT‐qPCR was performed in a 10 µL reaction volume with TB Green Premix Ex Taq II (RR820A, Takara, Japan) under the following processes: 95°C for 30 s, followed by 40 cycles of 95°C for 5 s and 60°C for 30 s, and melting curve analysis at 95°C for 10 s, 65°C for 60 s, and 95°C for 1 s. Relative mRNA levels of *OCLN* and *TNF* were calculated by the ^△△^Ct method with β‐actin as a reference. The primer sequences were: β‐actin (Forward: 5′‐CCTTCCCTCCTCAGATCATTGC‐3′, Reverse: 5′‐ATACTCCTGCTTGCTGATCCAC‐3′); *OCLN* (Forward: 5′‐CACGCTTGCCTGGGACAGAG‐3′, Reverse: 5′‐TCTGTATAGCCTCCGTAGCC‐3′); TNF (Forward: 5′‐CTCATCTACTCCCAGGTCCTCTTC‐3′, Reverse: 5′‐CGATGCGGCTGATGGTGTG‐3′).

### Statistical analysis

Statistical data was analyzed by R (v4.3.3) and GraphPad Prism (v10). All the experiment data are presented as the Mean ± SD. Wilcoxon rank‐sum test or *T* test was performed for comparisons between two groups. Significance was achieved when false discovery rate (FDR) < 0.05 or *p* < 0.05, where appropriate. Bioinformatic analysis was partially performed using the OmicStudio tools at https://www.omicstudio.cn/tool.

## AUTHOR CONTRIBUTIONS

**Xiaorou Qiu**: Writing—original draft; methodology; data curation; investigation; validation; formal analysis; software; visualization. **Chao Mu**: Methodology; investigation; writing—review and editing; validation; formal analysis; software; data curation; visualization; writing—original draft. **Jie Hu**: Validation; investigation; data curation. **Jiaxin Yu**: software; data curation; visualization. **Wenbo Tang**: Validation; data curation; investigation. **Yueli Liu**: Investigation. **Yongmei Huang**: Investigation. **Yixian Lu**: Data curation; investigation. **Peihua Tang**: Data curation; investigation. **Jingzhen Wu**: Visualization. **Zixuan Huang**: Investigation. **Xianlin Mei**: Investigation. **Huaguo Xiang**: Investigation. **Hao Lin**: Funding acquisition; project administration; resources; supervision. **Yi Qi**: Funding acquisition; project administration; resources; supervision. **Hui Luo**: Funding acquisition; project administration; supervision; resources. **Xuemeng Li**: Writing—review and editing; funding acquisition; project administration; resources; supervision; investigation; conceptualization; data curation.

## CONFLICT OF INTERESTS STATEMENT

The authors declare no conflicts of interest.

### ETHICAL STATEMENT

The study was approved (No. PJ2021‐113) by the Ethics Committee of the Affiliated Hospital of Guangdong Medical University, and informed consent was obtained from all participants.

## Supporting information


**Figure S1.** Characteristic metabolites analysis in centenarians (CE), lineal relatives of the centenarians (CE‐L), and control group of Elderly and Young.
**Figure S2.** KEGG enrichment analysis of metabolites among CE, CE‐L, Elderly and Young group.
**Figure S3.** Characteristic genera of gut microbiota in centenarians (CE), lineal relatives of the centenarians (CE‐L), and control group of Elderly and Young.
**Figure S4.** Characteristic genera of oral microbiota in centenarians (CE), lineal relatives of the centenarians (CE‐L), and control group of Elderly and Young.
**Figure S5.** The abundance of Serotonin, Indolelactic acid, 6‐Hydroxymelatonin, 3‐Methylindole, 3‐Indoleacrylate, L‐Tryptophan, and Quinolinic acid among CE, CE‐L, Elderly and Young group.
**Figure S6.** The CCK‐8 assay was used to detect the effects of 5‐MIAA on WI‐38 cells induced with concentrations of 50, 75, and 100 μM for 72 h.
**Figure S7.** CCK‐8 assay was used to test the optimal concentration of DSS and 5‐MIAA in Caco‐2 cells.

**Table S1.** General characteristics of study participants.
**Table S2.** 808 serum metabolites detected in the cohort.
**Table S3.** The list of the 47 centenarian‐specific serum metabolites.
**Table S4.** The physiological and cognitive functions of 145 centenarians.
**Table S5.** General characteristics of healthy and frail centenarians.

## Data Availability

The raw sequence data of the serum metabolome and 16S rRNA reported in this paper have been deposited in the Genome Sequence Archive [66] at the National Genomics Data Center [67], China National Center for Bioinformation/Beijing Institute of Genomics, Chinese Academy of Sciences (serum metabolome: OMIX009339, gut microbiota: CRA021552 and oral microbiota: CRA021555). These data are publicly accessible at https://ngdc.cncb.ac.cn/omix/release/OMIX009339, https://ngdc.cncb.ac.cn/gsa/browse/CRA021552 and https://ngdc.cncb.ac.cn/gsa/browse/CRA021555, respectively. The data and scripts used are saved in GitHub https://github.com/XuemengLi6758/Qiu2024iMeta. Supplementary materials (figures, tables, graphical abstract, slides, videos, Chinese translated version, and update materials) may be found in the online DOI or iMeta Science http://www.imeta.science/.
