# Supplementary material for: Serum metabolic and microbial profiling yields insights into promoting effect of tryptophan‐related metabolites for health longevity in centenarians
Source: Imeta. 2025 Apr 20;4(3):e70025. doi: 10.1002/imt2.70025 (PMC12130558; doi:10.1002/imt2.70025)
Supplement: Supplementary file 1 — Figure S1. Characteristic metabolites analysis in centenarians (CE), lineal relatives of the centenarians (CE‐L), and control group of Elderly and Young. Figure S2. KEGG enrichment analysis of metabolites among CE, CE‐L, Elderly and Young group. Figure S3. Characteristic genera of gut microbiota in centenarians (CE), lineal relatives of the centenarians (CE‐L), and control group of Elderly and Young. Figure S4. Characteristic genera of oral microbiota in centenarians (CE), lineal relatives of the centenarians (CE‐L), and control group of Elderly and Young. Figure S5. The abundance of Serotonin, Indolelactic acid, 6‐Hydroxymelatonin, 3‐Methylindole, 3‐Indoleacrylate, L‐Tryptophan, and Quinolinic acid among CE, CE‐L, Elderly and Young group. Figure S6. The CCK‐8 assay was used to detect the effects of 5‐MIAA on WI‐38 cells induced with concentrations of 50, 75, and 100 μM for 72 h. Figure S7. CCK‐8 assay was used to test the optimal concentration of DSS and 5‐MIAA in Caco‐2 cells. [file IMT2-4-e70025-s001.docx]

**Supporting information to**

**Serum metabolic and microbial profiling yields insights into promoting effect of tryptophan related metabolites for health longevity in centenarians**

**Running title:** Tryptophan metabolite 5-MIAA link to centenarian longevity

Xiaorou Qiu^1,2,3#^, Chao Mu^1,2#^, Jie Hu^1,4^, Jiaxin Yu^1,2^, Wenbo Tang^1,2^, Yueli Liu^1^, Yongmei Huang^1,5^, Yixian Lu^1,2^, Peihua Tang^1,2^, Jingzhen Wu^1,2^, Zixuan Huang^1,2^, Xianlin Mei^1,2^, Huaguo Xiang^3^, Hao Lin^6*^, Yi Qi^1,5*^, Hui Luo^1,5*^, Xuemeng Li^1,2*^

^1^Zhanjiang Key Laboratory of Human Microecology and Clinical Translation Research, the Marine Biomedical Research Institute of Guangdong Zhanjiang, Guangdong Medical University, Zhanjiang 524000, China

^2^Dongguan Key Laboratory of Stem Cell and Regenerative Tissue Engineering, the First Dongguan Affiliated Hospital, School of Basic Medical Sciences, Guangdong Medical University, Dongguan 523000, China

^3^The Medical Laboratory, Fuyong People's Hospital of Baoan District, Shenzhen 518000, China

^4^Institute of Cancer Prevention and Treatment, Harbin Medical University, Harbin 150000, China

^5^Southern Marine Science and Engineering Guangdong Laboratory (Zhanjiang), Zhanjiang 524000, China

^6^Suixi County People's Hospital, the Affiliated Hospital of Guangdong Medical University, Zhanjiang 524000, China

^#^These authors contributed equally: Xiaorou Qiu, Chao Mu

^*^Correspondence: lixuemeng@gdmu.edu.cn (Xuemeng Li), luohui@gdmu.edu.cn (Hui Luo), qiyi7272@gdmu.edu.cn (Yi Qi), linhao@gdmu.edu.cn (Hao Lin)

**Supplemental figure**


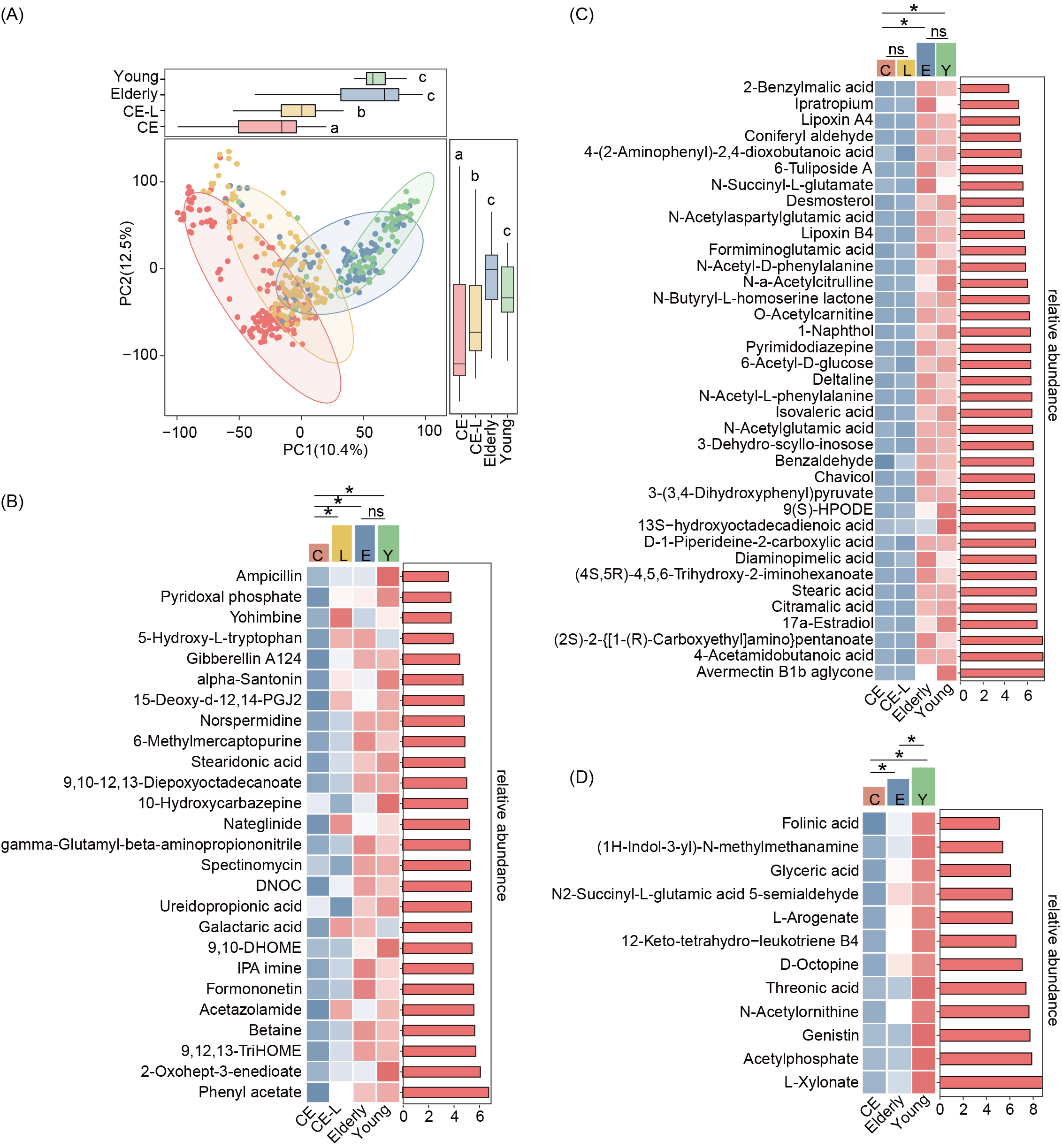


**Figure S1 Characteristic metabolites analysis in centenarians (CE), lineal relatives of the centenarians (CE-L), and control group of Elderly and Young.** (A) PLS-DA of serum metabolites in the study cohort under negative ion mode. (B-D) Characteristic metabolites decreased in centenarians (B), decreased in longevity families (C) and age-related decreases in metabolites (D).


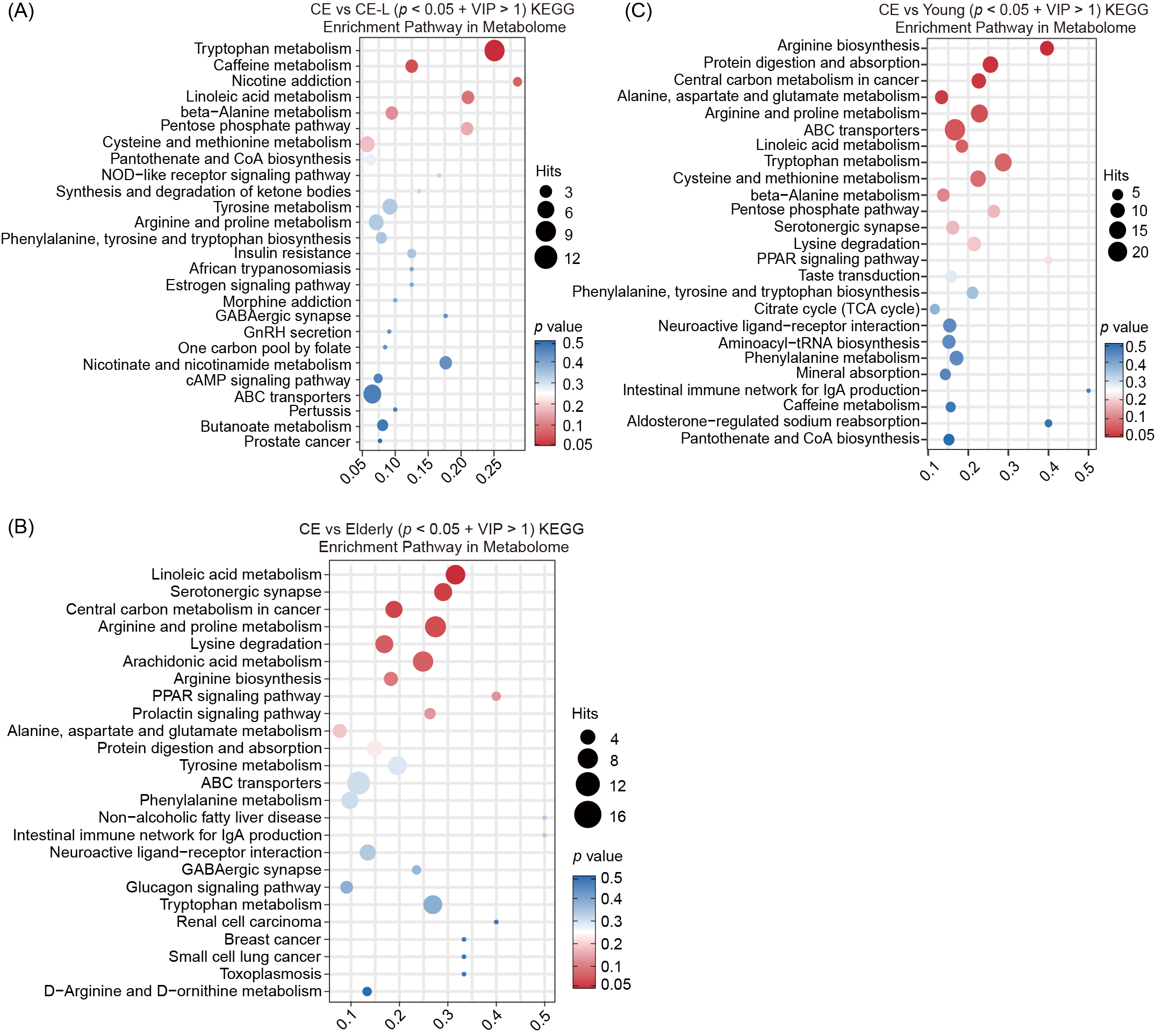


**Figure S2** **KEGG enrichment analysis of metabolites among CE, CE-L, Elderly and Young group.** (A-C) Top 25 metabolic pathways associated with differential metabolites in comparisons of CE vs CE-L (A), CE vs Elderly (B), and CE vs Young (C), respectively (VIP > 1, *p* < 0.05).


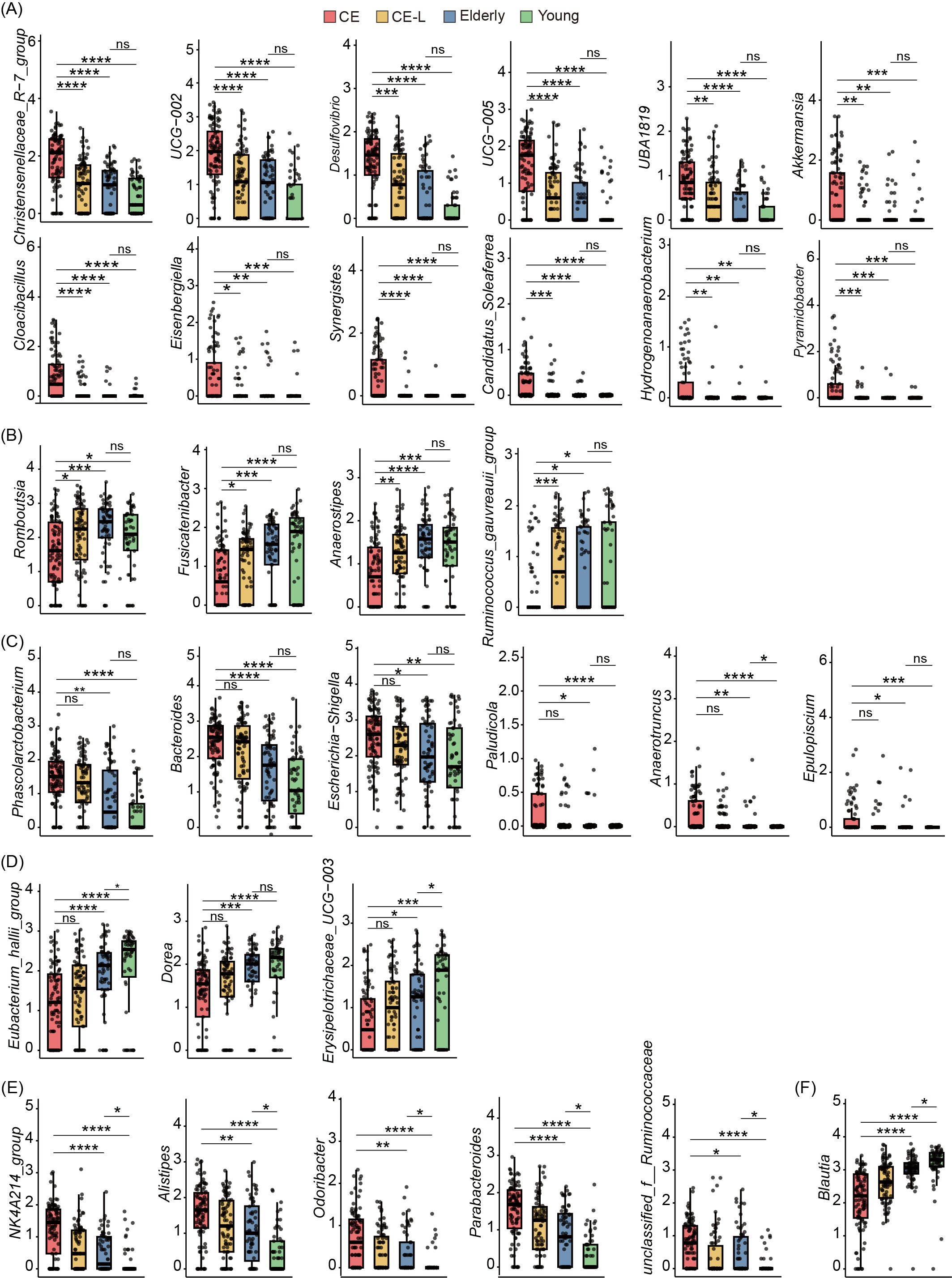


**Figure S3** **Characteristic genera of gut microbiota in centenarians (CE), lineal relatives of the centenarians (CE-L), and control group of Elderly and Young.** (A-B) Characteristic genera with centenarian signature: (A) demonstrating the genera enriched in centenarians and (B) showing the depleted genera in centenarians. (C-D) Characteristic genera with longevity family signature: (C) demonstrating the genera enriched in longevity family and (D) showing the depleted genera in longevity family. (E-F) Characteristic genera with aging signature: (E) demonstrating the gut genera enriched with aging, and (F) showing the genera depleted with aging. Statistical analysis was performed by Wilcoxon rank-sum test. * *p* < 0.05, ** *p* < 0.01, *** *p* < 0.001, **** *p* < 0.0001; ns: no significance (FDR adjustment).


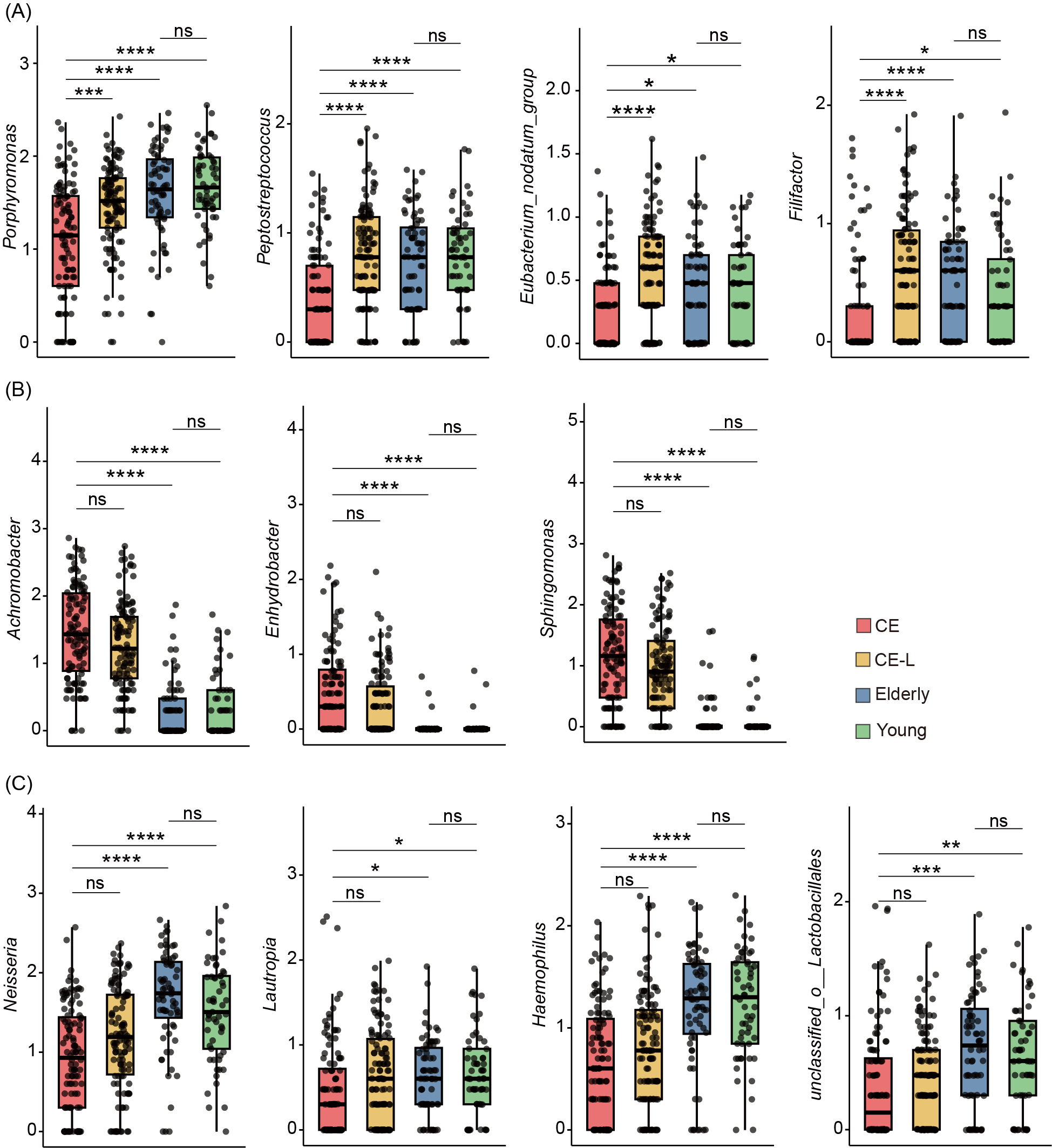


**Figure S4** **Characteristic genera of oral microbiota in centenarians (CE), lineal relatives of the centenarians (CE-L), and control group of Elderly and Young.** (A) The depleted genera in centenarians. (B-C) Characteristic genera with longevity family signature: (B) demonstrating the genera enriched in longevity family and (C) showing the depleted genera in longevity family. Statistical analysis was performed by Wilcoxon rank-sum test. * *p* < 0.05, ** *p* < 0.01, *** *p* < 0.001, **** *p* < 0.0001; ns: no significance (FDR adjustment).


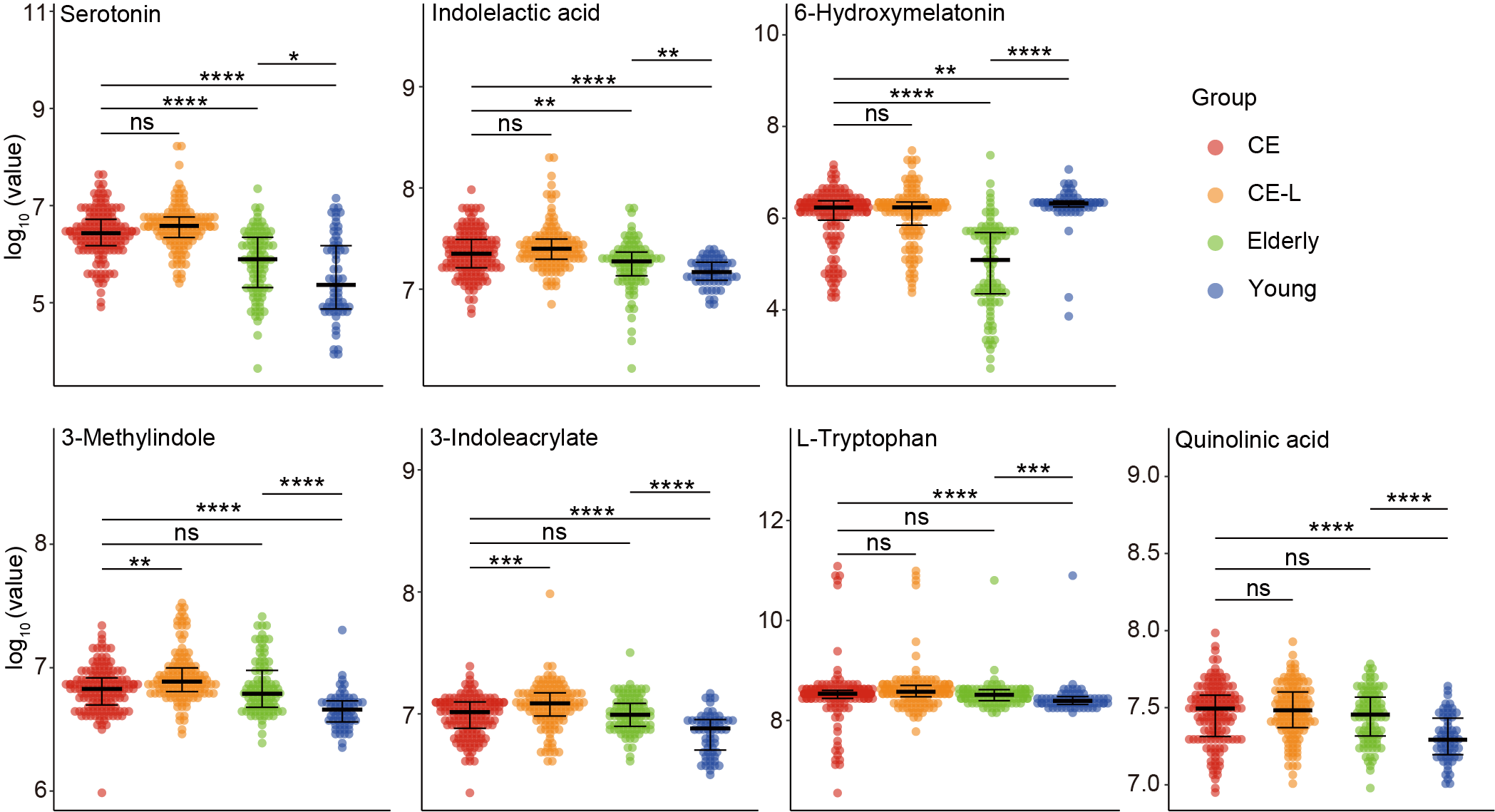


**Figure S5 The abundance of Serotonin, Indolelactic acid, 6-Hydroxymelatonin, 3-Methylindole, 3-Indoleacrylate, L-Tryptophan and Quinolinic acid among CE, CE-L, Elderly and Young group.** Statistical analysis was performed by Wilcoxon rank-sum test. * *p* < 0.05, ** *p* < 0.01, *** *p* < 0.001, **** *p* < 0.0001; ns: no significance (FDR adjustment).


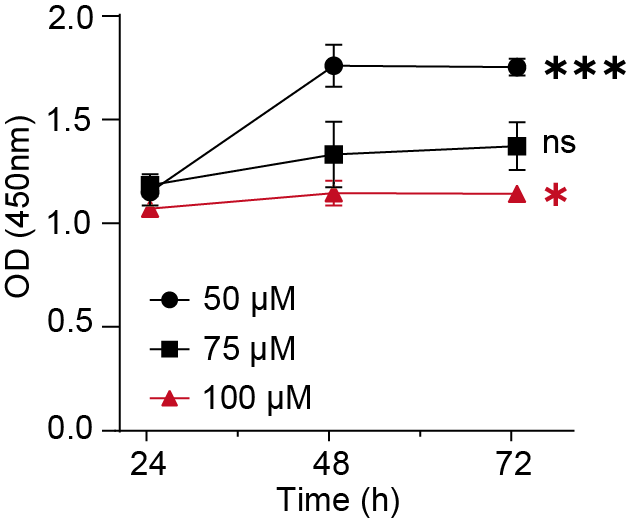


**Figure S6 The CCK8 assay was used to detect the effects of 5-MIAA on WI-38 cells induced with concentrations of 50, 75 and 100 μM for 72h.** Statistical analysis was conducted by comparing the OD values at 72h with those at 24h. Data are expressed as Mean ± SD. ns: no significance; * *p* < 0.05, *** *p* < 0.001.


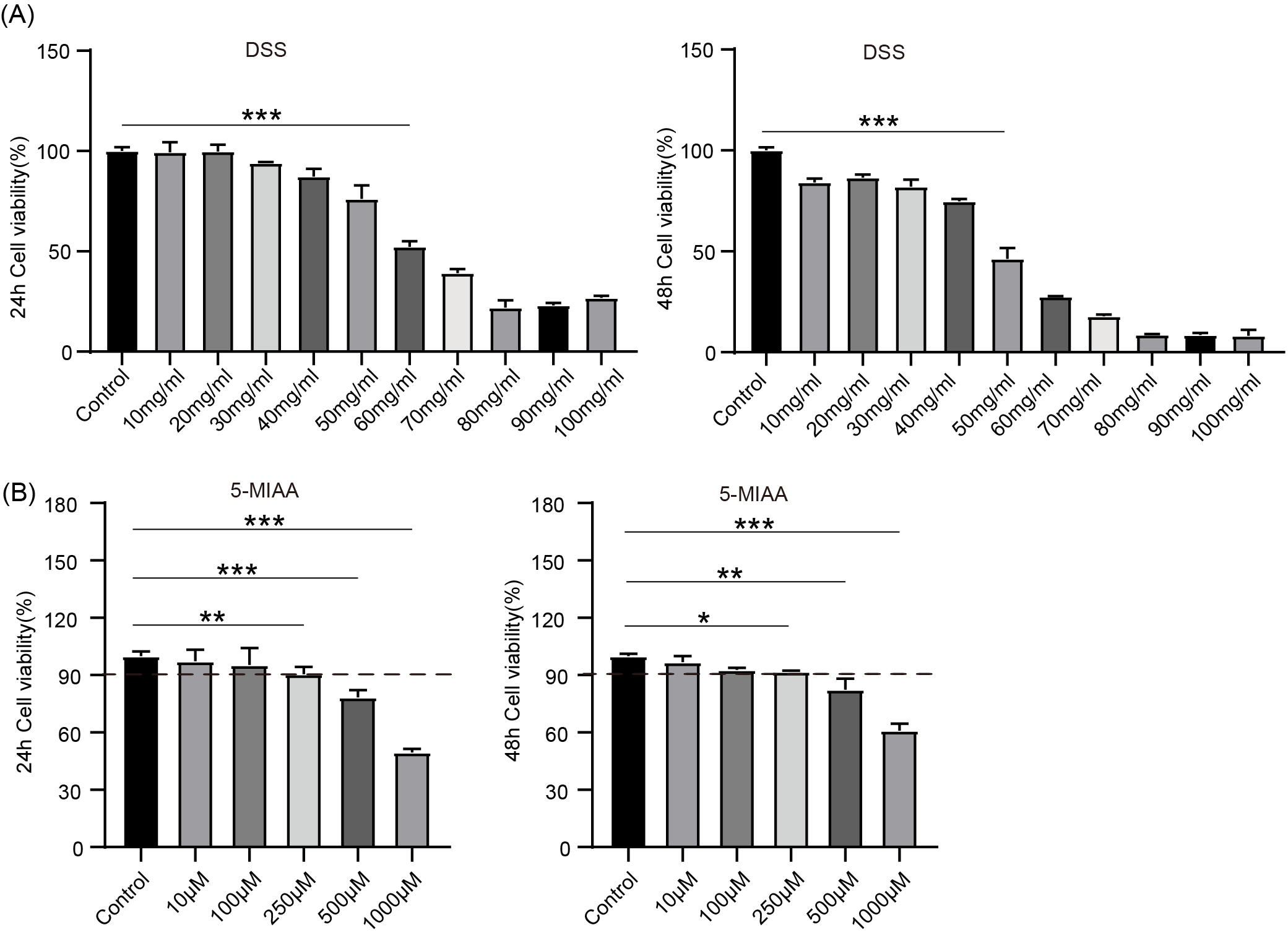


**Figure S7** **CCK8 assay was used to test the optimal concentration of DSS and 5-MIAA in Caco-2 cells.** (A) the cell viability of Caco-2 cells induced with different concentrations of DSS for 24 h and 48 h. (B) the cell viability of Caco-2 cells induced with different concentrations of 5-MIAA for 24 h and 48 h. Data are expressed as Mean ± SD. * *p* < 0.05, ** *p* < 0.01, *** *p* < 0.001.
